# Gene Regulatory Network of Human GM-CSF-Secreting T Helper Cells

**DOI:** 10.1155/2021/8880585

**Published:** 2021-07-03

**Authors:** Szabolcs Éliás, Angelika Schmidt, David Gomez-Cabrero, Jesper Tegnér

**Affiliations:** ^1^Unit of Computational Medicine, Center for Molecular Medicine, Department of Medicine Solna, Karolinska Institutet, ki.se Karolinska University Hospital & Science for Life Laboratory, 17176 Solna, Stockholm, Sweden; ^2^Mucosal & Salivary Biology Division, King's College London Dental Institute, London SE1 9RT, UK; ^3^Navarrabiomed, Complejo Hospitalario de Navarra (CHN), Universidad Pública de Navarra (UPNA), IdiSNA, 31008 Pamplona, Spain; ^4^Biological and Environmental Sciences and Engineering Division, King Abdullah University of Science and Technology (KAUST), Thuwal 23955–6900, Saudi Arabia; ^5^Biological and Environmental Sciences and Engineering Division, Computer, Electrical and Mathematical Sciences and Engineering Division, King Abdullah University of Science and Technology (KAUST), Thuwal 23955–6900, Saudi Arabia

## Abstract

GM-CSF produced by autoreactive CD4-positive T helper cells is involved in the pathogenesis of autoimmune diseases, such as multiple sclerosis. However, the molecular regulators that establish and maintain the features of GM-CSF-positive CD4 T cells are unknown. In order to identify these regulators, we isolated human GM-CSF-producing CD4 T cells from human peripheral blood by using a cytokine capture assay. We compared these cells to the corresponding GM-CSF-negative fraction, and furthermore, we studied naïve CD4 T cells, memory CD4 T cells, and bulk CD4 T cells from the same individuals as additional control cell populations. As a result, we provide a rich resource of integrated chromatin accessibility (ATAC-seq) and transcriptome (RNA-seq) data from these primary human CD4 T cell subsets and we show that the identified signatures are associated with human autoimmune diseases, especially multiple sclerosis. By combining information about mRNA expression, DNA accessibility, and predicted transcription factor binding, we reconstructed directed gene regulatory networks connecting transcription factors to their targets, which comprise putative key regulators of human GM-CSF-positive CD4 T cells as well as memory CD4 T cells. Our results suggest potential therapeutic targets to be investigated in the future in human autoimmune disease.

## 1. Introduction

CD4-positive T helper cells (Th) are crucial players in the immune system which exert their effects mainly by producing cytokines. CD4 T cell subsets are usually classified based on expression of “lineage-defining” transcription factors (TFs) as well as the signature cytokines they secrete [1]. However, the distinction is not clear-cut, since different signature cytokines can be expressed simultaneously and plasticity between subsets occurs [2, 3]. In view of the “classical” distinction of CD4 T cell subsets, particularly, Th1 and Th17 subsets are involved in the establishment of autoimmune diseases such as multiple sclerosis (MS) and the corresponding rodent model experimental autoimmune encephalomyelitis (EAE), which are thought to be driven by the T cell-released cytokines interleukin-17 (IL-17), interferon-*γ* (IFN-*γ*), IL-22, and granulocyte-macrophage colony-stimulating factor (GM-CSF). Of these, GM-CSF was determined as the key cytokine in EAE pathogenesis, since only knocking out GM-CSF (but neither IFN-*γ*, IL-17A, nor IL-17F) could completely protect the animals from induced EAE [4–6]. Furthermore, it has been demonstrated that specifically, the GM-CSF produced by autoreactive T cells was necessary for EAE induction, while T cell-produced IFN-*γ* and IL-17 were dispensable [7–9]. In line with these results, GM-CSF expression by Th cells was required for neuroinflammation in EAE, and even in the presence of IFN-*γ*- and IL-17A-producing Th cells, pathogenicity vanished upon loss of GM-CSF [10]. Importantly, in humans, the fraction of GM-CSF-positive (and IFN-*γ*-positive) cells within CD4 T cells was elevated in MS patients' cerebrospinal fluid compared to controls, while IL-17A-positive cell fractions were not strikingly different in these reports [11, 12]. The fractions of GM-CSF-positive and IFN-*γ*-positive cells were also increased in peripheral blood of MS patients in one report [13], but not in another [11]. A third study found a trend of increased IFN-*γ*-producing peripheral blood cells in MS patients, but among 12 different cytokines tested, only GM-CSF-positive cells were significantly increased in MS patients compared to controls [14]. Interestingly, the expanded Th subset-producing GM-CSF that was found in blood and CNS of MS patients could be diminished by disease-modifying therapy [14], suggesting GM-CSF-producing Th cells to be an attractive therapeutic target. Similarly, enhanced fractions of GM-CSF-producing CD4 T cells have been observed in synovial fluid of patients with juvenile arthritis along with the well-known enhanced GM-CSF levels in synovial fluid [15, 16]. Of note, targeting GM-CSF in MS or arthritis is subject to several ongoing clinical studies, highlighting the importance of this cytokine in these diseases [16]. Based on this cumulative evidence of the significance of GM-CSF-producing CD4 T cells in human autoimmune disease, understanding the factors driving and defining GM-CSF-positive T cells would be of utmost importance for targeting them therapeutically.

Beyond their established pathogenic role in autoimmune diseases, GM-CSF-producing Th cells have also been implicated in other inflammatory diseases. In sepsis, enhanced fractions of GM-CSF-producing T cells were associated with a poor outcome [17]. Notably, GM-CSF-producing Th cells have also been implicated in SARS-CoV-2 infection, especially in patients with a severe course of coronavirus disease 2019 (COVID-19) [18–20]. Due to the pleiotropic roles of GM-CSF in immune disease and lung inflammation, GM-CSF-targeting therapeutic approaches are currently explored in clinical trials to treat COVID-19 [21].

Murine CD4 T cell populations expressing IL-17A and GM-CSF have been observed and termed “pathogenic Th17” cells because they have the potential to induce EAE [22–24]. However, only one of these studies showed coexpression of both cytokines on the single-cell level [24]. Although a T cell can express GM-CSF simultaneously with other cytokines such as IFN-*γ*, a “GM-CSF-only-producing” murine T cell subset was also proposed and associated with enhanced encephalitogenic activity over IL-17 and IFN-*γ*-producing T cells in EAE [9]. The existence of a corresponding separate “GM-CSF-only” human T cell subset has also been proposed [25], because a substantial subset of *ex vivo*-restimulated human GM-CSF-positive CD4 T cells produces GM-CSF in the absence of any other classical Th1, Th2, and Th17 lineage-defining cytokines (such as IFN-*γ*, IL-4, and IL-17), transcription factors, or surface markers [11, 26]. Furthermore, GM-CSF-producing CD4 T cells are induced by different sets of cytokines compared to other Th cell subsets [11, 25, 27]. In fact, GM-CSF and IL-17A expression by human CD4 T cells has been found to be mutually exclusive on the single-cell level [11] or at least substantially less frequent than coexpression of IFN-*γ* and GM-CSF [11, 26, 27]; also, IL-4 coproduction with GM-CSF was negligible [11]. A more recent study [14] has extended the analysis of cytokine coexpression in human PBMCs upon Phorbol 12-myristate 13-acetate (PMA) and ionomycin restimulation to a range of 13 different cytokines by the use of mass cytometry and also included MS patients along with controls. Of these cytokines tested, cytokines typical for Th2, Th17, Th22, or Tfh cells were not coproduced by GM-CSF-positive Th cells, while a substantial fraction coproduced the Th1-cytokine IFN-*γ*, as well as TNF*α* and IL-2 [14].

Despite the importance of GM-CSF-producing T cells, there is no specific marker to distinguish such cells from others to date. Although combinations of the presence and absence of nonexclusive surface markers have been useful to delineate “GM-CSF-only” cells [11], the fraction of GM-CSF-producing cells that also produces other cytokines such as IFN-*γ* is excluded by this approach. Furthermore, the driving factors for GM-CSF production remain unclear. Together, these observations suggest that the characterization of human GM-CSF-positive CD4 T cells isolated based on their functional profile (GM-CSF production) rather than using the “classical” Th1 and Th17-like phenotypic markers may enable the identification of factors regulating GM-CSF production in CD4 T cells. Hence, in order to understand the regulators and molecular patterns defining human GM-CSF-positive CD4 T cells, we isolated those cells actively secreting GM-CSF from human peripheral blood *ex vivo* by cytokine “capture” assay, starting with bulk CD4 T cells. We then studied their transcriptome by RNA-sequencing (RNA-seq). Studying a single data type like mRNA expression separately may not be sufficient for identification of all regulatory factors, since TFs themselves are often regulated by posttranscriptional modifications, intracellular translocation, or cobinding with other TFs, rather than by changes in their gene expression. Thus, we assessed in parallel the DNA accessibility of the same samples in order to gain a global picture of putative TF binding patterns and enabling integration with the expression of regulated target genes on the RNA level. Due to the limited number of primary, *ex vivo*-isolated GM-CSF-positive human T cells, we employed a recently described highly sensitive method assay for transposase-accessible chromatin using sequencing (ATAC-seq) [28] to study DNA accessibility from 50,000 cells per replicate. As a control, we used the respective GM-CSF-depleted (GM-CSF-negative) fraction derived from the capture assay procedure. Since GM-CSF-positive cells (which may also coproduce additional cytokines and hence may represent “activated” T cells) may differ from GM-CSF-negative CD4 T cells merely by containing largely reduced fractions of naïve cells, we furthermore undertook RNA-seq and ATAC-seq profiling of several control cell populations from the same donors, namely, naïve CD4 T cells and memory CD4 T cells. As an additional control, we studied bulk CD4 T cells without a capture assay procedure.

This study hence reveals molecular patterns of GM-CSF-positive CD4 T cells as well as those shared with memory, naïve, or bulk CD4 cells. To our knowledge, this is the first study of global molecular signatures of GM-CSF-positive CD4 T cells derived *ex vivo* without restimulation. Although this population does not only contain “GM-CSF-only” cells, but may include cells producing other cytokines, the *ex vivo* isolation based on active GM-CSF secretion results in a pure population of cells producing GM-CSF versus cells not producing GM-CSF. Besides serving as a control, we furthermore provide a novel resource of ATAC-seq and RNA-seq data of human primary naïve, memory, and bulk CD4 T cells from several human healthy donors. A large body of knowledge exists on molecular signatures and regulation of human naïve and memory T cells [29]. This encompasses large consortium efforts to map human memory and naïve CD4 T cell subsets' transcriptomes and epigenomes including chromatin accessibility [30], albeit these authors did not use the ATAC-seq method. Recently, few reports using ATAC-seq for T cells have been published and the impactful results support the power of the methodology. Many of these studies focus on murine CD8 T cell differentiation and exhaustion [31–35], and several recent studies also comprise ATAC-seq on CD4 T cells [10, 28, 36–40]. However, none of these works studied all the types of CD4 T cell subsets that we analyzed here.

Through interpreted, integrative analysis of mRNA expression and DNA accessibility data from primary human CD4 T cell subsets, we provide novel gene regulatory networks underlying GM-CSF production as well as the memory phenotype in human CD4 T cells and we propose novel key TFs regulating these cells. The enrichment of the identified genes for human immune system diseases and especially MS for GM-CSF-positive cells underlines the clinical relevance of our data, which may be exploited in a multitude of basic and applied immunology studies in the future.

## 2. Materials and Methods

### 2.1. Ethics Statement

Peripheral blood mononuclear cells were freshly isolated from anonymized healthy donor buffy coats purchased from the Karolinska University Hospital (Karolinska Universitetssjukhuset, Huddinge), Sweden. Research was performed according to the national Swedish ethical regulations (ethical review act, SFS number 2003:460).

### 2.2. Experimental Methods

#### 2.2.1. PBMC and T Cell Isolation

Human peripheral blood mononuclear cells (PBMCs) were isolated using Ficoll-Paque gradient centrifugation from buffy coats according to standard procedures. In brief, buffy coats were diluted in PBS, layered on Ficoll-Paque (GE Healthcare), and centrifuged (1200 × g, 20 min, without break). Subsequently, the PBMC ring was collected. PBMCs were washed with PBS (450 × g, 10 min) and monocytes were depleted by plastic adherence in RPMI 1640 medium containing GlutaMAX (Life Technologies, Thermo Fisher Scientific) and 10% (*v*/*v*) heat-inactivated fetal bovine serum (FBS) (Gibco Performance Plus certified; Thermo Fisher Scientific) for 60–80 minutes at 37°C, 5% CO_2_. Platelets were removed by centrifugation (200 × g, 5–10 min, 20°C, 4 times). Subsequently, human naïve, memory, and total CD4 T cells were isolated negatively (“untouched”) in parallel by magnetic-activated cell sorting (MACS) from each donor. The following MACS kits were used according to the instructions from the manufacturer (Miltenyi Biotec): human naïve CD4^+^ T cell isolation kit II, human memory CD4^+^ T cell isolation kit II, and human CD4^+^ T cell isolation kit II. The purity of naïve, memory, and total CD4 T cells was controlled by flow cytometry (see below). Cells were counted with the Countess Automated Cell Counter (Invitrogen), and viability (determined by Trypan blue stain) was 96.5 ± 1.5% (mean ± SD). T cells were cultured at 37°C and 5% CO_2_ in serum-free X-Vivo 15 medium (Lonza) supplemented with GlutaMAX (Gibco), unless otherwise stated. T cells were rested overnight before sample preparation for RNA-seq, ATAC-seq, and GM-CSF secretion assay (see below). 6 healthy male anonymized donors (aged 35.7 ± 8.5 years, mean ± SD; range 22–44 years) were used for molecular profiling.

#### 2.2.2. GM-CSF Secretion Assay (Capture Assay)

Total CD4 T cells were isolated and rested as above, before capturing GM-CSF-producing cells using the "GM-CSF Secretion Assay-Cell Enrichment and Detection Kit (PE), human" (Miltenyi Biotec) according to the manufacturer's instructions with the following modifications and details. 65–150 × 10^6^ (98.3 × 10^6^ ± 33.7 × 10^6^, mean ± SD) unstimulated purified CD4 T cells were centrifuged (300 × g, 10 min), X-Vivo 15 medium was removed completely, and cells were washed with 15 ml MACS buffer (0.5% human serum albumin (HSA) and 2 mM EDTA in PBS, 4°C). Cells were resuspended in ice-cold RPMI 1640 medium including GlutaMAX (Life Technologies, Thermo Fisher Scientific) and 10% (*v*/*v*) FBS (Gibco Performance Plus certified, heat inactivated; Thermo Fisher Scientific). GM-CSF catch reagent was added, mixed, and incubated for 5 minutes on ice. The GM-CSF secretion period was performed by adding prewarmed (37°C) RPMI 1640 medium (containing GlutaMAX and 10% FBS) to a cell density of 1 × 10^6^ cells/ml, under continuous rotation (10 rpm orbital mixing) of the cells for 45 minutes at 37°C, 5% CO_2_. Labeling cells with GM-CSF Detection Antibody (Biotin) and Anti-Biotin-PE and magnetic labeling with Anti-PE MicroBeads UltraPure were performed as per manufacturer's instructions, and cells were washed in 50 ml MACS buffer. Cells were resuspended in 3 ml MACS buffer, cell suspensions were filtered with 30 *μ*m Filcon strainer (BD Biosciences), and magnetic separation was performed on LS columns (Miltenyi Biotec) according to standard protocols. For donor A, the GM-CSF+ eluate was passed over a second MS column following the LS column procedure, but since a second column did not increase purity but led to loss of cells (data not shown), for all other donors, the GM-CSF+ eluate from the first LS column was used for subsequent analyses. The GM-CSF− fraction from the flow-through was passed over a second column (except for donor A) to increase purity of the negative fraction. The yield of GM-CSF+ cells was 1.2 ± 0.6% of CD4 T cells (mean ± SD) and purity was controlled by flow cytometry (see below).

#### 2.2.3. Flow Cytometry

**Figure 1:**
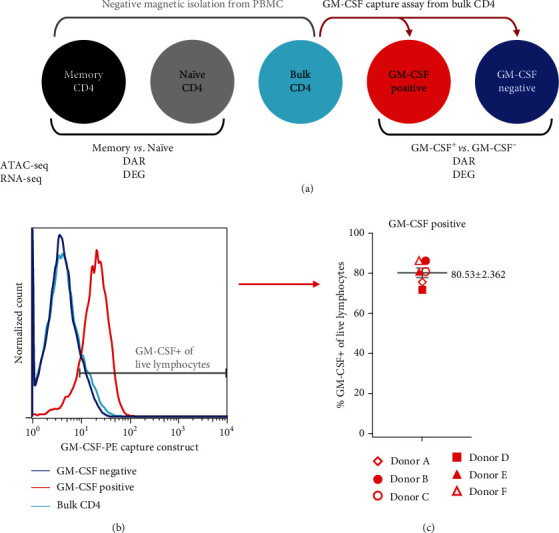
Experimental setup and quality control for human CD4 T cell transcriptome and chromatin accessibility analysis. (a) Human PBMCs for each donor were split in three fractions, and memory CD4 T cells, naïve CD4 T cells, and bulk (total) CD4 T cells were isolated in parallel by negative (untouched) magnetic isolation (cell purities; see Supplementary Figure S1). From bulk CD4 T cells, GM-CSF-secreting cells (GM-CSF positive) were captured and the negative fraction was used as the corresponding GM-CSF-negative population. The five indicated cell populations were used for molecular profiling by ATAC-seq and RNA-seq. Differentially accessible DNA regions (DAR) and differentially expressed genes (DEG) were determined for the comparison of memory versus naïve CD4 T cells and for GM-CSF-positive versus GM-CSF-negative CD4 T cells, respectively. (b, c) High purity of isolated (captured) GM-CSF+ CD4 T cells was confirmed by flow cytometry. The histograms show the signal of the GM-CSF capture construct for the indicated cell populations, pregated on live singlet lymphocytes based on forward scatter, side scatter, and pulse width. Here, bulk CD4 T cells represent an aliquot taken after labeling with the GM-CSF-PE capture construct, but without further capture assay procedure. (b) shows a representative donor and (c) shows summarized data for *n* = 6 donors from 4 independent experiments (mean and SEM are indicated in grey). Each donor is represented by a symbol; same symbol shape (but filled or unfilled) indicates donors processed within the same experiment.

Purity of MACS-isolated naïve CD4 T cells, memory CD4 T cells, and total CD4 T cells was verified by surface staining using the following antibodies (all against the human proteins): CD4-PerCP (clone SK3, BD Biosciences), CD45RA-FITC (clone T6D11, Miltenyi Biotec), CD45RO-PE (clone UCHL1, BD Biosciences), CD19-APC (clone HIB19, BD Biosciences), and CD8-eFlour450 (clone OKT8, eBioscience). Staining was performed in the dark with antibody dilutions in FACS buffer (PBS with 0.5% HSA) for 15 minutes at 20°C. Single-stained PBMC or T cell samples were used as compensation controls. Cells were washed once with PBS, resuspended in FACS buffer, and acquired on a CyAn ADP 9-Color Analyzer (Beckman Coulter). Compensation was performed with the CyAn software (Summit) tool. Purity of GM-CSF+ and GM-CSF− samples after GM-CSF secretion assay from bulk CD4 T cells was assessed by measuring the fraction of PE-labeled (GM-CSF-positive) cells. Cell purities and gating strategy are shown in Figure 1 and Supplementary Figure S1. We also confirmed in independent experiments (including PMA and ionomycin restimulation) that the PE label in GM-CSF-captured cells correlated well with the GM-CSF signal based on intracellular staining with an anti-GM-CSF antibody (clone BVD2-21C11, APC conjugated, Miltenyi Biotec; data not shown). Flow cytometry data analysis and visualization were performed using the FlowJo software v7.6.5 (Tree Star), and exported percentage values were plotted using GraphPad Prism v7.02 (GraphPad Software Inc.).

#### 2.2.4. ATAC-Seq Sample Preparation

Nuclear isolation, tagmentation, and PCR amplification was carried out according to Buenrostro et al. [28]. In brief, 50,000 cells per replicate were transferred to 0.2 ml tubes and centrifuged (500 × g, 6 min, 4°C) and the supernatant was removed. Cells were washed with PBS (500 × g, 6 min, 4°C) and lysed in lysis buffer (10 mM Tris-HCl, pH 7.4; 10 mM NaCl; 3 mM MgCl_2_; and 0.1% IGEPAL CA-630) to isolate nuclei. 3 technical replicates (50,000 cells each) were processed in parallel for each sample (except donor A, 2 technical replicates). Nuclei were washed in PBS (500 × g, 6 min, 4°C) and resuspended in Transposase Reaction mix. Transposition was carried out at 37°C for 30 minutes, followed by clean up using the QIAGEN MinElute Reaction Clean Up Kit according to the manufacturer's instructions (QIAGEN). PCR amplification using reagents from the Nextera DNA Sample Preparation Kit (Illumina) and barcoding of replicates were performed with reaction conditions and index primers as described in [28]. 20 different index primers were used and distributed across replicate samples and donors in a balanced way to control for potential batch effects. The PCR product was cleaned up using the QIAGEN MinElute Reaction Clean Up Kit. Subsequently, gel size selection was performed by gel electrophoresis (1.8% (*w*/*v*) certified low-melt agarose (Bio-Rad) in 1x UltraPure Tris-Acetate-EDTA (TAE) buffer including SYBR Safe DNA Gel Stain (Invitrogen, Thermo Fisher Scientific), with a free well between each of the samples) and DNA in the size range of 150–230 bp was excised using surgical blades. Replicate samples were allocated to the gels in a balanced way regarding donor and experimental condition to control for potential batch effects. Resulting DNA libraries were purified using QIAGEN MinElute Gel Extraction Kit according to the manufacturer's instructions (QIAGEN).

Size distribution of ATAC-seq sequencing libraries was determined on a 2100 Bioanalyzer Instrument (Agilent Technologies) using the Agilent DNA High Sensitivity Kit according to the manufacturer's instructions. Libraries were quantified by real-time PCR on a StepOnePlus detector system (Applied Biosystems) using the KAPA Library Quantification Kit (KAPA Biosystems). Sequencing was performed on an Illumina HiSeq 2500 instrument (Illumina) with a single-read setting and read length of 42 bp.

#### 2.2.5. RNA-Seq Sample Preparation

Cells were centrifuged (1000 × g, 5 min, 20°C), washed with PBS, and lysed in QIAzol Lysis Reagent (QIAGEN) by vortexing and incubating for 5 minutes at 20°C. Lysates were stored at −80°C until RNA extraction. RNA was extracted with the miRNeasy Micro Kit (QIAGEN) according to the manufacturer's instructions. RNA concentration was determined on a NanoDrop 2000 spectrophotometer (Thermo Fisher Scientific), and RNA quality was controlled on a 2100 Bioanalyzer Instrument (Agilent Technologies) using an Agilent RNA 6000 Pico Kit. RNA integrity numbers (RIN) were 8.5 ± 0.3 (mean ± SD). Libraries were prepared in one batch with the TruSeq Stranded mRNA HT Kit (Illumina) with Dual Index Adapters and Ambion ERCC Spike-In Control (Thermo Fisher Scientific). Samples were allocated to sequencing indexes and lanes in a balanced fashion to control for potential batch effects (7–8 samples/lane). Library concentration was determined on a Qubit 2.0 Fluorometer (Thermo Fisher Scientific). The library size and quality were measured on a 2100 Bioanalyzer Instrument (Agilent Technologies) using an Agilent High Sensitivity DNA Kit. Libraries were quantified with the KAPA Library Quantification Kit (KAPA Biosystems). Sequencing was performed on a HiSeq 2500 Sequencing Platform (Illumina; High Output Run) with 76 nt paired-end reads.

### 2.3. Computational Methods

#### 2.3.1. Preprocessing of Sequencing Data

BCL base call files were demultiplexed and converted to FASTQ files using bcl2fastq version 2.17.1.14. For quality control, FastQC version 0.11.5 was used.

FASTQ files from the ATAC-seq experiments were trimmed from adapters, and low-quality bases using scythe version 0.991. and sickle version 1.33. FASTQ files from the ATAC-seq experiments were aligned to the human genome version hg38 using bowtie version 2.3.0 with the ‘--very-sensitive' option. After alignment, BAM files of ATAC-seq experiments were filtered to eliminate: duplicates (samtools rmdup), alignments with a mapping score below 10, and alignments that are not mapped to chromosome 1-22, chromosome X, or chromosome Y. ATAC-seq peaks were called using the ‘findPeaks' script from the HOMER suite (version 4.9.1) with the ‘-style factor' option [41]. ATAC-seq peaks were assigned to a gene using the ‘annotatePeaks' script from the HOMER suite.

FASTQ files from the RNA-seq experiments were aligned using STAR version 2.5.2b. Indexes for RNA-seq alignment were created using the gencode version 25 annotation file. RNA-seq alignment was run with STAR's built-in adapter trimming option (‘--clip3pAdapterSeq AGATCGGAAGAGCACACGTCTGAACTCCAGTCAC AGATCGGAAGAGCGTCGTGTAGGGAAAGAGTGTA') and its built-in counting option (‘--quantMode'). Only genes with more than 1 count per million in at least 3 samples were included in the downstream RNA-seq analysis.

#### 2.3.2. Generation of Consensus Peak Set

In order to generate a set of comparable features (genomic regions) for read counting and quantifying differential accessibility from the ATAC-seq data, a set of consensus peaks was generated in two subsequent steps: (1) generation of consensus peaks on the technical replicate level: first, the peaks that appeared in at least two technical replicates (out of a total of three, except one donor with a total of two technical replicates) with at least 75% reciprocal overlap were selected. Then, these selected regions were partitioned into disjoint nonempty subsets so that each element is contained in precisely one subset. Only the partitions appearing in at least two replicates were retained, and afterwards, adjacent regions were merged. A bed file resulting from these steps is herein referred to as a sample and (2) next, all bed files containing each set of technical replicate level consensus regions were concatenated and the presence of each region was counted within each experimental sample; one occurrence corresponds to one donor (biological replicate) and one cell type (experimental condition). Only the regions appearing in at least four samples were kept (*n* = 5 is the number of biological replicates in the smallest group regarding experimental condition). Regions having a distance of 42 bases or less between them were subsequently merged (the number 42 corresponds to the sequencing read length in bases). Afterwards, reads were counted using the featureCounts tool [42] with the criterion that at least half of the read had to overlap with a feature to be assigned to that feature.

#### 2.3.3. Calculation of Differential Expression and Differential Accessibility

Differential expression and accessibility was calculated using the edgeR [43] library version 3.18.1 from Bioconductor. Donors (biological replicates) and cell types (experimental conditions) were used as explanatory variables in the generalized linear models. ATAC-seq data were normalized to length and GC content by conditional quantile normalization (CQN) [44]. Comparisons were made between GM-CSF-positive CD4 T cells and GM-CSF-negative CD4 T cells or between memory and naïve CD4 T cells (see Figure 1(a)). The cutoff to call differentially expressed genes (DEGs) or differentially accessible regions (DARs) was FDR < 0.05 and >25% fold change (in the direction of either up- or downregulation, that is either 1.25 or 0.75 fold change).

#### 2.3.4. Footprinting

Footprinting was carried out using the Wellington algorithm [45], i.e., the wellington_footprints.py script from the pyDNase library version 0.2.5 with the following settings: -fp 6,41,1 -sh 7,36,1 -fdr 0.01 -fdriter 100 -fdrlimit -30 -A. The footprint occupancy score (FOS) for each footprint was calculated using the pyDNase library as described in [46]. For subsequent network reconstruction, we considered only footprints with a FOS smaller than the following threshold: median (FOS) + [median (FOS)–minimum (FOS)].

#### 2.3.5. Network Reconstruction

A directed network was reconstructed by combining information from the ATAC-seq and RNA-seq data in two subsequent steps: (1) identifying source nodes: peaks were ranked based on their combined measure of significance and direction of differential accessibility [−log_10_ (FDR) × sign (log_2_ (fold change))]. Peaks containing footprints were scanned for TF binding motifs using the TRANSFAC database [47]. An enrichment score was calculated to identify TFs with binding sites enriched in differentially accessible peaks, using tools similar to gene set enrichment analysis (GSEA) [48]. In detail, random permutation was performed on the ranked list of peaks to assess how probable it is to observe at least the same enrichment by chance (*P* value) [49]. After multiple testing correction, TFs with FDR < 0.05 were selected and the normalized enrichment score (NES) was obtained. Only those source nodes (TFs) that were detectably expressed on the RNA level (according to a minimal RNA-seq filtering rule) were considered. Of these, most fell into the class of highly expressed genes (HEGs) according to [50]. (2) identifying target nodes: target nodes are defined as peaks with an assigned gene. The selection criteria were that (i) the peak contains a footprint with a binding motif of the source node (TF) and (ii) the peak or the assigned gene has to be differentially accessible or differentially expressed, respectively.

To assign an importance measure to the source nodes in networks generated as above, the PageRank [51] of the network nodes was calculated after inverting the directionality of all edges in the network (only for the purpose of this computation). After this computation, the nodes with high PageRank values (higher than the 99th percentile of all node values within a given network) were selected from both the GM-CSF and memory network, and afterwards, their values were investigated in each of the two networks.

## 3. Results and Discussion

GM-CSF-positive CD4 T cells are enriched in MS patients and play a crucial role in EAE; nevertheless, the factors driving and markers defining those cells are largely unknown. To better understand the features and regulatory networks of GM-CSF-positive CD4 T cells, we therefore studied the transcriptional profiles and chromatin accessibility of these cells. *In vitro*-differentiated GM-CSF-producing cells comprise several subsets [27] and are likely to differ from those generated *in vivo*. Further, *ex vivo* restimulation with strong artificial stimuli such as PMA and ionomycin—which is usually necessary to reach sufficient signal strength for detection by intracellular cytokine staining—drastically alters the transcriptome of T cells [52–54]. Hence, we aimed to isolate GM-CSF-producing cells *ex vivo* in an as much as possible unmanipulated state by GM-CSF secretion assay, “capturing” and isolating those cells that actively secrete GM-CSF (experimental setup; see Figure 1(a)), here defined as GM-CSF-positive cells. The capture assay was performed starting from highly purified CD4 T cells derived from human peripheral blood (purity 97.3 ± 0.6%, mean ± SEM, Supplementary Figure S1A). As controls, we used the respective GM-CSF-negative fraction from the isolation procedure, as well as the bulk CD4 T cells before any capture assay procedure. The latter should, given the low fraction of GM-CSF-positive cells, be very similar to the GM-CSF-negative fraction and hence allows for estimation of the effects arising from the capture assay procedure. The purity of GM-CSF-positive and GM-CSF-negative fractions was assessed by flow cytometry (Figures 1(b) and 1(c)) and the yield of isolated GM-CSF-positive cells was 1.2 ± 0.6% (mean ± SD) of CD4 T cells. To measure the transcriptome and DNA accessibility from limited cell numbers, we employed highly sensitive next-generation sequencing (NGS) methods (RNA-seq and ATAC-seq, respectively). Since cytokine-secreting cells may differ from naïve cells due to a memory-like phenotype and a large fraction of CD4 T cells are naïve (mean ± SEM, 42.3 ± 5.8% in the donors used here; see Supplementary Figure S1A), we further profiled highly purified naïve and memory CD4 T cells from the same donors (Figure 1(a) and Supplementary Figure S1B-D).

Altogether, we obtained DNA accessibility and transcriptome data from highly purified *ex vivo*-derived human naïve CD4 T cells, memory CD4 T cells, bulk CD4 T cells, and GM-CSF-positive and the corresponding GM-CSF-negative CD4 T cells. To enable paired analysis within a donor, these cell populations were isolated in parallel within a donor, for 6 donors in total. DNA accessibility and mRNA data were obtained in parallel from the same samples allowing for matched integration of the data.

### 3.1. Unique and Shared DNA Accessibility and Gene Expression Signatures of GM-CSF-Positive and Memory CD4 T Cells

**Figure 2:**
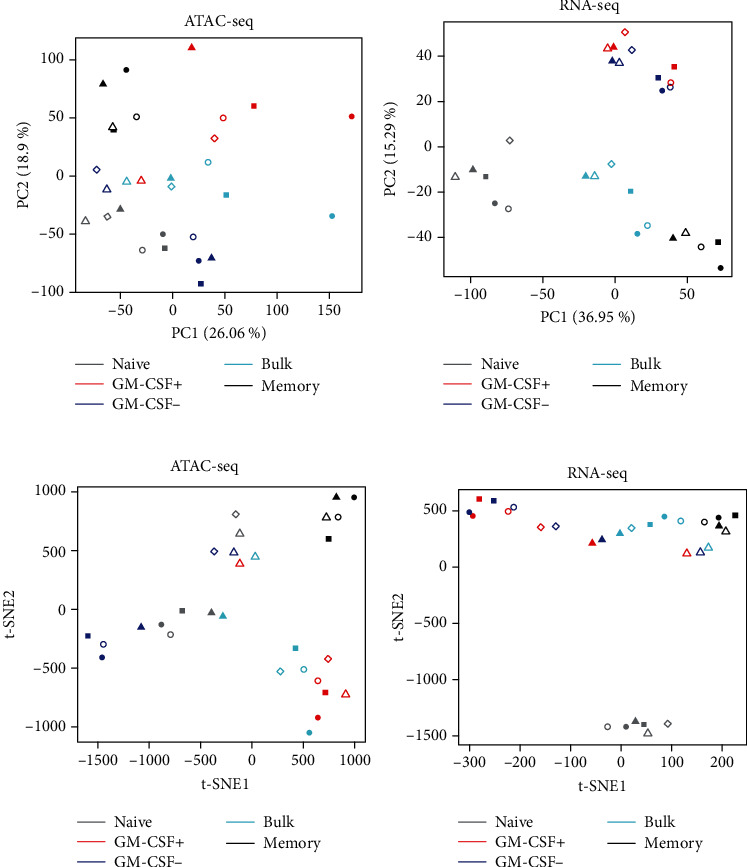
Explorative analysis of ATAC-seq and RNA-seq samples. (a) Principal component analysis (PCA) was carried out on CQN-normalized ATAC-seq data given as log_2_(RPKM + 1) centered by mean subtraction for each feature (genomic region). PC1 and PC2 are shown, along with the % variation explained. Symbol colors indicate the given cell populations and symbol shapes and fillings represent individual donors (*n* = 5–6 donors) as in Figure 1(c). Data from technical replicates were pooled before analysis. (b) PCA for RNA-seq data given as log_2_(FPKM + 1) centered by mean subtraction for each feature (gene). Labels as in (a). (c, d) t-SNE dimensionality reduction visualization of (c) ATAC-seq and (d) RNA-seq data, processed and labeled as in (a) and (b), respectively.

We studied the above-described five different CD4 T cell populations by RNA-seq and ATAC-seq. To minimize potential batch effects due to technical factors, the library preparations and sequencing runs were designed in such a way that donors, cell populations, and (for ATAC-seq) technical replicates were distributed in a balanced fashion. It is also worth noting that only donors of the same gender were studied here (male, aged 35.7 ± 8.5 years, mean ± SD), which may be important since it was recently shown that gender was the largest source of variation explaining chromatin accessibility in primary human CD4 T cells measured by ATAC-seq [36]. That study further discovered novel elements escaping X chromosome inactivation and affecting immune genes [36]. To assess which factors explained most of the variability between the samples under study here, we performed principle component analysis (PCA). Indeed, for both data types, there was a grouping of the samples based on the cell subset, outweighing donor or experimental variation (Figures 2(a) and 2(b)) and confirming the quality of our samples and data. Notably, for RNA data, the cell populations were generally more distinct from each other than for DNA accessibility data. However, the GM-CSF-positive and corresponding GM-CSF-negative fractions appeared relatively similar to each other in the PCA performed on RNA data, while PCA results from ATAC-seq data were closer to the expected pattern, that is, bulk CD4 T cells appearing “between” GM-CSF-positive and GM-CSF-negative populations (Figures 2(a) and 2(b)). The difference between RNA-seq and ATAC-seq data with respect to separation of GM-CSF-positive and negative cells may indicate that the capture assay procedure imposes distinct changes on the transcriptome, highlighting the importance of using correspondingly treated controls to determine differential expression. In contrast, changes in DNA accessibility appeared more robust towards changes due to the experimental procedure at least within the experimental time frame under study, although the distinction of the other groups was generally less apparent with ATAC-seq data. The advantage of PCA is that the displayed distances between samples along the axes can directly be interpreted but it is not suited to reduce all the data variability to only two dimensions. Indeed in our analysis, the first two PCs in the two-dimensional space only explained about 50% of the variation in the data. Therefore, we also used another dimensionality reduction method to explore the sample-to-sample relationships, namely, t-distributed stochastic neighbor embedding (t-SNE) [55]. t-SNE allows for visualization of sample-to-sample similarity in two dimensions, and furthermore, in contrast to PCA, it is a nonlinear dimensionality reduction algorithm and it is especially suited for capturing local relationships. The t-SNE results (Figures 2(c) and 2(d)) generally confirmed the results of the PCA analysis (Figures 2(a) and 2(b)), that is, the groups (cell types) being more distinct in RNA data than ATAC-seq data, with the exception of GM-CSF-positive and GM-CSF-negative cells.

**Figure 3:**
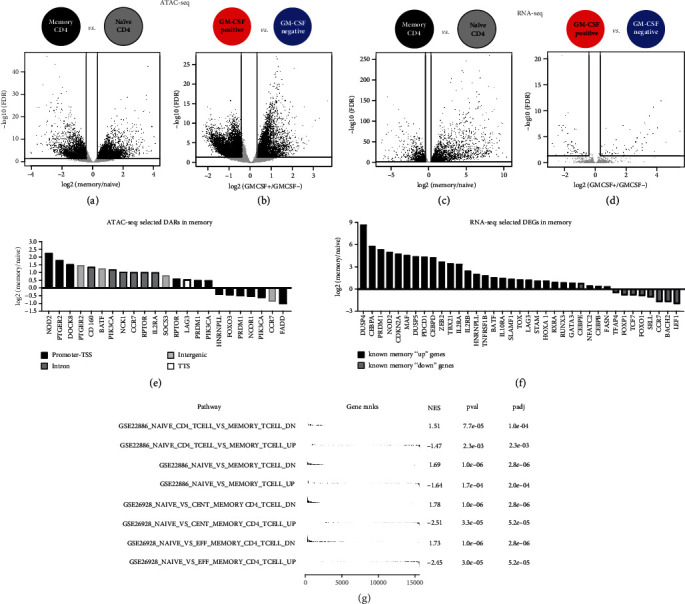
Differential chromatin accessibility and gene expression in memory versus naïve CD4 T cells as well as in GM-CSF-positive versus negative cells. (a, b) ATAC-seq data were CQN normalized and differential accessibility between the indicated cell population comparisons was calculated ((a) memory *vs.* naïve CD4 T cells; (b) GM-CSF-positive *vs.* negative CD4 T cells). Volcano plots show each consensus peak as a single dot, and lines indicate the threshold for calling a DAR (FDR < 0.05 and >25% fold change); DARs are depicted in black. (c, d) Differential gene expression was calculated from RNA-seq data for cell population comparisons as in (a, b). Lines indicate the threshold for calling a DEG (FDR < 0.05 and >25% fold change); DEGs are depicted in black. (e) A selection of DARs in memory *vs.* naïve was studied for being assigned to genes known to play a role in T cell memory. log_2_(fold change (memory/naïve)) for selected DARs are plotted, and colors indicate the category that the respective region is assigned to (TSS: transcription start site; TTS: transcription termination site). (f) Known T cell memory “up” (black) or T cell memory “down” (grey) genes based on previous literature were selected if differentially expressed (DEG in memory *vs.* naïve) in the present data. log_2_(fold change (memory/naïve)) for these selected DEGs is plotted; values > 0 represent upregulation and values < 0 represent downregulation in memory T cells. Colors indicate whether the gene was previously described to be “up” or “down” in memory T cells. (g) Gene set enrichment analysis (GSEA) using gene sets from published transcriptome data featuring naïve and memory T cell subsets (GSE accession numbers as displayed) and a ranked gene list of our memory versus naïve T cell data. Ranking was based on −log_10_(*P* value) × sign(log_2_(fold change (memory/naïve))). Gene sets containing both human naïve and memory T cell data were retrieved from the MSigDB database [48]. NES: normalized enrichment score; pval: *P* value; padj: FDR.

We defined significantly differentially accessible DNA regions (DARs) and significantly differentially expressed genes (DEGs) in GM-CSF-positive CD4 T cells or in memory CD4 T cells. To do so, we specifically analyzed the signatures of GM-CSF-positive versus GM-CSF-negative CD4 T cells, as well as the profiles of memory versus naïve CD4 T cells (Figures 1(a), 3(a), 3(b), 3(c), and 3(d)). We used generalized linear models based on the negative binomial distribution (edgeR) [43] to determine differential expression and accessibility, and we called DEGs and DARs, respectively, based on combined FDR and fold change cutoffs. We called 16571 DARs in GM-CSF-positive CD4 T cells (compared to corresponding GM-CSF-negative cells; Figure 3(b)) and 13705 DARs in memory CD4 T cells (compared to naïve CD4 T cells; Figure 3(a)). On the transcriptome level, we called 124 DEGs in GM-CSF-positive and 5383 DEGs in memory CD4 T cells (Figures 3(c) and 3(d)). The relatively low number of DEGs in GM-CSF-positive cells is in agreement with the PCA and t-SNE data (Figures 2(b) and 2(d)) and may suggest that combination with ATAC-seq data drastically improves the possibility to define molecular signatures specific to GM-CSF-positive *ex vivo-*captured cells.

Next, we studied the DARs and DEGs in more detail. We first focused on the signatures of memory CD4 T cells, which are well studied in the literature [29] and hence enabled to assess the biological quality of our data, besides providing a new NGS dataset of human primary memory and naïve CD4 T cells. DARs and DEGs defined in memory cells are shown in Supplementary Figure S2, along with their molecular patterns in the other cell types under study. Accessible regions (consensus peaks) were annotated to the major categories promoter-TSS (46.6% of all peaks), intron (26.8%), and intergenic (18.7%), followed by exon (4.1%) and TTS (3.8%). Regions differentially accessible (DARs) in memory cells showed a similar distribution across annotated categories (44.7% in promoter-TSS, 26.9% intron, 20.4% intergenic, 3.9% exon, and 4.1% TTS). We next extracted those memory-specific DARs that were assigned to a gene from a list of genes known to be involved in T cell memory as compiled by Durek and colleagues [30]. Several of the DARs in memory cells were assigned to such known memory-associated genes, about half (12 of 23) of those selected regions were falling in the promoter-TSS region and about a quarter (6 of 23) were assigned to intronic regions (Figure 3(e)). Furthermore, we assessed a selected subset of memory-related genes that were shown to be up- or downregulated on the RNA level in memory T cells [30]. The majority of these genes were DEGs in memory cells in our data, notably up- or downregulated almost exclusively (36 of 37 studied genes; 97%) in the expected direction (Figure 3(f)), validating our data. In addition, we confirmed the quality of the memory T cell data by performing gene set enrichment analysis (GSEA) using several published transcriptome datasets comprising naïve and memory T cell subsets. Indeed, genes described to be up- or downregulated in memory (versus naïve) CD4 T cells in these published datasets were significantly enriched on the expected ends of the ranked gene list from our novel memory versus naïve T cell dataset (Figure 3(g) and Supplementary Figure S3).

### 3.2. The Molecular Signature of GM-CSF-Positive CD4 T Cells

**Figure 4:**
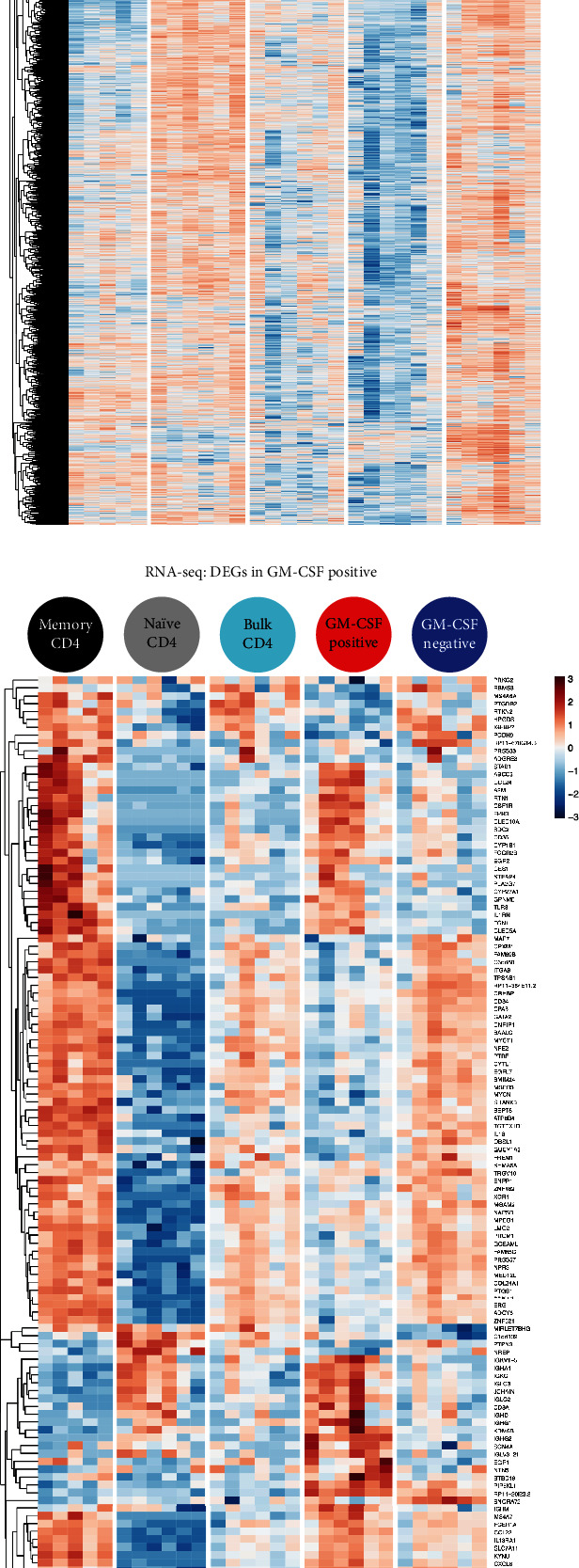
Expression profile and DNA accessibility signatures of human GM-CSF-positive CD4 T cells. (a) Differentially accessible DNA regions (DARs) in GM-CSF-positive versus GM-CSF-negative CD4 T cells are plotted as a heat map showing their accessibility in all the five cell populations studied. CQN normalized log_2_(RPKM + 1) is represented by the color scale, indicating accessibility (blue: low, red: high). Data were row scaled and clustered (Euclidean distance, complete linkage clustering). (b) Differentially expressed genes (DEGs) in GM-CSF-positive versus GM-CSF-negative CD4 T cells are plotted as a heat map showing their expression in all the five cell populations studied. Gene expression is displayed as log_2_(FPKM + 1) with blue indicating low and red indicating high expression according to the color scale. Data were row scaled and clustered (Euclidean distance, complete linkage clustering). (c) The mean of log_2_(fold change) of GM-CSF+/GM-CSF− cell populations using the median intensity values from CyTOF measurements are shown, using CD4 T cell-gated PBMC data from Wong et al. [64] and designated as “CyTOF” (grey bars). For the genes corresponding to the proteins measured in CyTOF, the log_2_(fold change) of GM-CSF+/GM-CSF− cell populations from the RNA-seq data of this study is plotted and labeled as “RNA” (red).

Next, we focused on the signatures of GM-CSF-positive cells by studying the respective DARs and DEGs in more detail. All the DARs in GM-CSF-positive cells are displayed in Figure 4(a) with the color scale representing accessibility. A substantial fraction of DARs in GM-CSF-positive cells displayed a similar pattern in memory cells, while naïve cells and GM-CSF-negative cells were most distinct from GM-CSF-positive cells (Figure 4(a)). In bulk CD4 T cells, DARs defined in GM-CSF-positive cells showed heterogeneity between donors (Figure 4(a)), which may reflect the variability in the fraction of memory and naïve cells within bulk CD4 T cells depending on the donor (Supplementary Figure S1A). Importantly, several DARs displayed a unique accessibility pattern in GM-CSF-positive cells distinct from other cell populations under study (Figure 4(a)). These data show that GM-CSF-positive cells can be assigned a specific pattern of accessible DNA regions that distinguish them from other CD4 T cell subsets, and that may contribute important information about regulation of GM-CSF-positive cells. Different DNA accessibility can functionally affect the status of a cell by, for example, modifying expression of genes regulated through these regions.

Next, we studied the DEGs defined in GM-CSF-positive cells by analyzing their expression in GM-CSF-positive cells along with the other CD4 T cell subsets under study. Like it was observed for the DARs, DEGs in GM-CSF-positive cells shared a large part of the RNA signature with memory cells but also displayed distinct patterns and differed largely from naïve cells and GM-CSF-negative and bulk CD4 T cells (Figure 4(b)). Despite the general similarity of cells treated with the capture assay regarding the global transcriptome (Figures 2(b) and 2(d)), subsetting on the DEGs defined between GM-CSF-positive and negative cells with stringent statistical cutoffs visualized clearly the differences in these “signature genes” for those cell types (Figure 4(b)). Importantly, subsetting on these genes that were defined as DEGs in GM-CSF-positive versus corresponding negative cells (that is, without considering the bulk CD4 T cell samples) also clearly showed the expected similarity of bulk CD4 T cells and GM-CSF-negative CD4 T cells (Figure 4(b)) that was not apparent in the RNA-seq PCA or t-SNE using all genes (see above; Figures 2(b) and 2(d)), confirming that the selected GM-CSF-positive cell “signature” DEGs are likely not affected by the capture assay and column procedure.

DEGs in GM-CSF-positive cells comprised several genes with a well-known role in T cells such as *EGR2*, *CXCL8*, and *CXCR5* along with multiple genes with an unknown role in T cells and potentially regulating GM-CSF-positive cells (Figure 4(b)). *CSF2RB* (encoding for the high-affinity receptor subunit for IL-3, IL-5, and GM-CSF) was not among the DEGs in GM-CSF-positive cells, and *CSF2RB* expression being lower (albeit not passing the significance threshold) in GM-CSF-positive than negative cells makes a technical artifact of isolation of cells binding GM-CSF through this receptor unlikely (Figure 4(b)).

It should be mentioned that while captured GM-CSF-positive cells displayed high purity regarding captured secreted GM-CSF and CD4 T cell marker positivity (Figures 1(b) and 1(c) and Supplementary Figure S1), this represents an enrichment of GM-CSF-producing cells versus cells not producing GM-CSF but it does not mean that the cells do not coexpress other cytokines typical for other Th subsets. Albeit not passing the significance threshold for being called a DEG in GM-CSF+ cells, relatively higher expression of *IFNG* mRNA in GM-CSF-positive cells versus GM-CSF-negative cells (Supplementary Figure S4A) is in accordance with our and others' previous findings from flow or mass cytometry of T cells from healthy donors as well as MS patients demonstrating coexpression of IFN-*γ* and GM-CSF on the single-cell level in some, but not all, GM-CSF-producing human CD4 T cells or clonal populations thereof [11, 14, 15, 26, 27, 56]. Also in line with the majority of these studies on human T cells regarding a lack of coexpression of GM-CSF and IL-17, *IL17A* and *IL17F* expression was below the detection limit in GM-CSF-positive cells (but also in any other cell population under study; Supplementary Figure S4A). Besides IFN-*γ*, TNF*α* and IL-2 proteins have also been found to be frequently coexpressed in GM-CSF-positive Th cells upon PMA + ionomycin restimulation [14], and as for *IFNG*, also *TNF* was slightly higher expressed in GM-CSF-positive versus negative cells, although not statistically significant (Supplementary Figure S4A). Importantly, it has to be noted that in human T cells restimulated *ex vivo* with PMA and ionomycin to detect cytokines by intracellular staining and cytometry as in the above studies, the fractions of TNF*α*-, IL-2-, and IFN-*γ*-producing Th cells are generally high (for example, up to 90% of all Th cells producing TNF*α*, up to 75% of Th cells producing IL-2, and up to 40% of Th cells producing IFN-*γ*) while cytokines typical for Th2, Th17, Th22, or Tfh cells are generally low (<5% of Th cells being positive for the respective cytokines) [14]. Consequently, coproduction of IL-2, TNF*α*, or IFN-*γ* protein is generally likely for any T cell subset restimulated with PMA and ionomycin. Nevertheless, due to the importance of IFN-*γ* and GM-CSF in the context of MS, we studied intracellular protein expression of GM-CSF and IFN-*γ* (requiring PMA and ionomycin restimulation) in bulk CD4 T cells of the donors used in this study. 49.98 ± 4.61% (mean ± SEM) of the GM-CSF-positive CD4 T cells coexpressed IFN-*γ* (Supplementary Figure S4B), although it remains unknown how this is influenced by the PMA and ionomycin restimulation needed for this analysis procedure *per se*. In this context, it is notable that strong restimulation by PMA and ionomycin, as needed in single-cell cytokine protein studies, may cause expression of abundant T cell cytokines such as IL-2 protein even in purified naïve Th cells after only 3 to 5 hours of stimulation (unpublished observation and [57]) that may not be truly present *in vivo* without artificial restimulation, as naïve Th cells are not expected to actively produce cytokines. Also on the global transcriptome level, PMA and ionomycin stimulation for only 30 minutes to 3 hours was shown to drastically alter the T cell gene expression signature [52–54]. Hence, to identify the *ex vivo* gene expression signature of GM-CSF-positive cells, we isolated GM-CSF-producing CD4 T cells without PMA and ionomycin restimulation in all our NGS profiling studies, which results in a low yield of cells yet represents cells in a state without artificial restimulation and hence preserving their gene signature as much as possible in the *ex vivo* state. While the low cell yield did not allow for parallel capture of other cytokines or protein analysis in the captured cells in these donors, the above-described cytokine mRNA analyses as well as parallel intracellular cytokine staining in bulk CD4 T cells from the same donors suggest that *IFNG* may be expressed in GM-CSF-positive, but also GM-CSF-negative, Th cells. However, it is noteworthy that expression of many cytokines on the mRNA level is rather low if the cells are not artificially restimulated *ex vivo*. Along these lines, many cytokine mRNAs were below or close to the detection limit and, hence, excluded from further differential expression analysis (Supplementary Figure S4A). Although many cytokine mRNAs were lowly expressed and did not pass the detection threshold, we nevertheless analyzed mRNA counts of relevant T cell cytokines in the cell populations under study even when expressed very lowly (Supplementary Figure S4A). Of the cytokines passing the detection threshold (*IFNG*, *TNF*, *IL13*, *IL17C*, *CXCL8*, and *GZMB*), only *CXCL8* (encoding for IL-8) was differentially expressed in GM-CSF-positive versus negative cells, while all except *IL13* were differentially expressed in memory versus naïve T cells. It should be noted that *CSF2* mRNA, which encodes for GM-CSF, was lowly expressed in all samples (RNA-seq counts close to the detection limit), which may be explained by rapid mRNA decay conferred by the adenine- and uridine-rich elements (ARE) in the GM-CSF promoter—AREs in fact have been discovered in the *CSF2* gene which codes for a particularly unstable transcript [58–60]. Importantly, considering the instability of *CSF2* mRNA and relatively low expression levels, our approach of isolating GM-CSF protein–secreting cells is likely to be more suitable to define signatures of *ex vivo*-derived GM-CSF-positive cells, as opposed to, for example, single–cell RNA-seq of mixed T cell populations. Strikingly, a recent study [61] performing single-cell RNA-seq of immune cells from MS patients confirmed that, despite the known importance of GM-CSF-producing Th cells in MS pathogenesis [11, 13, 14], *CSF2* mRNA was not detectable in that study, neither in the blood nor in cerebrospinal fluid samples [61]. Hence, signatures derived from our study designating GM-CSF-positive human T cells may be useful to identify such cells based on their signature in RNA studies where *CSF2* cannot be used as a suitable marker.

To our knowledge, there is no other comparable dataset available that studied the signatures of purified GM-CSF-secreting cells without PMA/ionomycin restimulation. Thus, the RNA expression signatures defined in the GM-CSF-positive cells in this study could not be directly validated externally in an independently published transcriptome dataset. Nevertheless, we strived to confirm the expression signature from GM-CSF-positive captured cells by comparing with a completely independent dataset and experimental setup. A recent report [62] provides RNA-seq data of GM-CSF-, IFN-*γ*-, and IL-17-producing captured cells from healthy donors; however, PMA and ionomycin restimulation renders it difficult to directly compare these data to ours. Interestingly, the authors [62] observed a large overlap of the gene expression signature between the cells producing either cytokine and, importantly, also to (PMA + ionomycin-stimulated) naïve CD4 T cells. This suggests that the strong stimulatory effect of PMA and ionomycin may largely dominate the gene expression signatures, which is supported by several studies that showed a large influence of even short (30 minutes to 3 hours) PMA plus ionomycin stimulation on the global transcriptome of human Th cells [52–54]. In addition, capture assay and flow cytometry-based cell sorting as used by Al-Mossawi et al. [62] may cause rapid changes to the transcriptome. Indeed, studies of the transcriptome after diverse cell isolation procedures have revealed that different isolation procedures have an impact on the global transcriptome [54, 63]. Nevertheless, we compared the published data with our transcriptome data to determine whether GM-CSF-positive cells may be closely related to cells producing GM-CSF or other cytokines in that study. However, PMA and ionomycin restimulation (or other differences in experimental setup) seemed to largely determine the sample-to-sample similarity, as all the samples correlated more strongly based on dataset than based on cell type (data not shown). Thus, we compared the similarity of the different cell types after batch normalization, and while naïve T cells were similar between the two datasets, the overall transcriptome patterns were not distinguishable based on cytokine expression, neither within the published dataset nor across datasets (Supplementary Figure S4C). This suggests that cytokine-producing CD4 T cell subsets have a largely similar transcriptome and that cell restimulation and isolation procedures dominate the global gene expression patterns and render it difficult to compare between studies. To further confirm the DEGs in GM-CSF-positive cells in external data and their relevance on the protein level, we asked whether the RNA expression pattern of captured GM-CSF-positive T cells generally agreed with the respective proteins in GM-CSF-positive T cells defined by intracellular cytokine staining. To explore overlap of as many as possible markers, we studied a mass cytometry (CyTOF) dataset [64] which comprises staining of human PBMCs with CD4 and GM-CSF along with other markers measured on the protein level. In pregated CD4-positive T cells of this CyTOF dataset, we gated on GM-CSF+ cells and GM-CSF− cells and determined the relative expression of other available protein markers in these populations. We compared the up- or downregulation of these protein markers in gated GM-CSF+ versus GM-CSF− cells [log_2_ (fold change) of the median signal intensity of the two cell populations] with the up- or downregulation of the corresponding protein-coding transcripts of these markers in isolated GM-CSF-positive versus GM-CSF-negative cells from our data [log_2_ (fold change) of mRNA expression]. As a result, 16 of the 24 markers (two-thirds) had a fold change with identical directions in the CyTOF and RNA-seq data (Figure 4(c)). According to a binomial distribution, the probability of observing this or greater concordance between the two datasets by chance is 7.6%. This needs to be acknowledged considering that those markers that do not concur might be regulated by protein internalization from the surface, such as the well known for CD3 [65] that was downregulated in the CyTOF data but barely affected in the mRNA data. Also, the total abundance of certain proteins may be regulated on the posttranscriptional level, as transcript levels cannot always predict protein abundance [66, 67]. In addition, PMA and ionomycin stimulation prior to intracellular cytokine staining may have affected some of these markers. Overall, we concluded that the expression profile of the GM-CSF-positive captured T cells matches well with the profile of independently characterized human GM-CSF-positive T cells.

### 3.3. GM-CSF-Positive CD4 T Cell Transcript Signatures and Chromatin Accessibility Are Associated with Autoimmune Diseases, Especially MS

**Figure 5:**
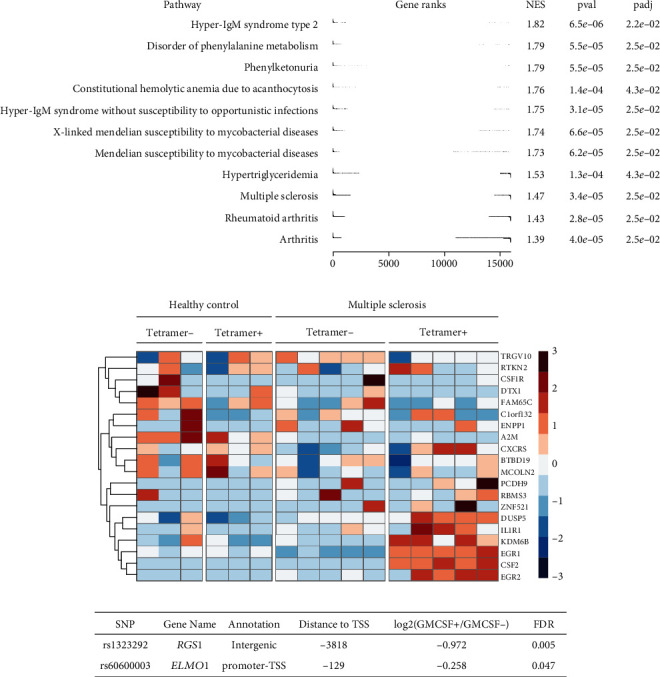
GM-CSF-positive CD4 T cell signatures are associated with autoimmune diseases, especially MS. (a) Gene set enrichment analysis is shown using genes ranked by the –log_10_(FDR) × sign(log_2_(GM − CSF+/GM − CSF−)) function, and the Open Targets database [68] was used to provide gene sets associated with diseases. NES: normalized enrichment score; pval: *P* value; padj: FDR. (b) The heat map represents the row-scaled log_2_(RPKM + 1) expression values from RNA-seq data of CD4 T cells from MS patients or healthy controls (data from [26]). Groups are separated based on disease status (MS or healthy) and myelin antigen reactivity (reactive: tetramer+). Genes are selected as those identified in the current RNA-seq study as differentially expressed between GM-CSF-positive versus GM-CSF-negative cells and having detectable expression (log_2_(RPKM + 1) > 0) in at least 4 samples in the data from [26]. (c) Of the 110 established non-MHC MS susceptibility variants [70], two SNPs mapped to consensus peaks from the current ATAC-seq study. Information about these SNPs is shown in the table, along with the differential accessibility analysis in GM-CSF-positive versus GM-CSF-negative CD4 T cells.

Next, we asked whether the gene expression pattern of GM-CSF-positive cells was enriched for any diseases by exploring the Open Targets Platform [68]. We ranked the detected genes based on their differential expression in GM-CSF-positive versus negative cells using the −log_10_ (FDR) × sign (log_2_ (fold change)) function for ranking and calculated enrichment scores for diseases. Indeed, among the few significantly enriched diseases (FDR < 0.05), the autoimmune diseases MS and rheumatoid arthritis were represented (Figure 5(a)). Notably, for both diseases, GM-CSF targeting is in clinical trials [16], supporting the relevance of our data. The data also showed enrichment for several other diseases related to the immune system, infection, or metabolism, although it should be noted that some of these disease gene sets contained only few elements (genes) and may thus be less relevant than MS or rheumatoid arthritis, which comprised a large number of genes (Figure 5(a) and Supplementary Table S1A). When performing the same enrichment analysis for the ranked gene list from memory versus naïve CD4 T cells, a large number of diseases were significantly enriched including many diseases involving the immune system, as expected (Supplementary Table S1B).

Due to the relevance of GM-CSF-positive T cells in MS, we studied in more detail whether DEGs identified in GM-CSF-positive cells displayed altered expression in MS patients' T cell samples. T cells recognizing peptides derived from myelin proteins as autoantigen and being activated and migrating to the CNS are thought to be crucial mediators of inflammation in MS [69]. Cao and colleagues have performed RNA-seq of expanded CD4 T cells derived from MS patients and healthy controls, with two subsets of samples each containing those autoreactive to antigenic peptides derived from myelin (tetramer-positive) versus tetramer-negative T cells [26]. The authors discovered that myelin-reactive T cells from patients with MS displayed strongly enhanced production of IFN-*γ*, IL-17, and GM-CSF compared to those isolated from healthy controls, which instead secreted more anti-inflammatory IL-10 [26]. We studied whether these cells would also display altered expression of the genes we defined as signature genes of GM-CSF-positive cells. Indeed, a subset of these genes was down- and another subset was upregulated in myelin-reactive T cells from MS patients (Figure 5(b)), potentially identifying genes relevant to the disease pathogenesis. Here, the T cells displayed detectable *CSF2* mRNA encoding for GM-CSF, perhaps due to the *in vitro* stimulation and expansion of these T cells. Remarkably, only myelin-reactive cells from MS patients expressed high levels of *CSF2* (Figure 5(b)). Interestingly, this *CSF2* expression pattern strongly resembled the expression patterns of several of the GM-CSF-positive T cell signature DEGs defined here, namely, *DUSP5*, *IL1R1*, *KDM6B*, *EGR1*, and *EGR2* (Figure 5(b)), which may be interesting candidates to explore in the future regarding their role in GM-CSF-positive T cells and MS. While this analysis confirmed the relevance of the GM-CSF-positive T cells' RNA-seq data in MS, we next analyzed whether also the ATAC-seq data could reveal information on those DNA regions most relevant for GM-CSF-positive T cells in the context of MS. To do so, we asked whether the peaks called from our ATAC-seq analysis contained any SNPs associated with MS. Interrogating a list of non-MHC MS susceptibility variants comprising 110 established risk variants from the International Multiple Sclerosis Genetics Consortium [70, 71], we identified two SNPs mapping to the accessible DNA peaks (considering all consensus peaks) from our study. These SNPs were assigned to the protein-coding genes *Regulator Of G Protein Signaling 1* (*RGS1*) and *Engulfment And Cell Motility 1* (*ELMO1*) genes (Figure 5(c)). Importantly, the regions containing these SNPs were significantly differentially accessible (FDR < 0.05) in GM-CSF-positive versus GM-CSF-negative cells (Figure 5(c)), suggesting a putative role of these regions in T cell-mediated MS pathogenesis. It is tempting to speculate that the epigenetic signature defined here may be useful as a surrogate signature to define GM-CSF-producing cells, especially in the context of recently available single-cell ATAC-seq methodologies [72, 73] and the instability of *CSF2* mRNA leading to failure to detect GM-CSF-producing cells based on *CSF2* mRNA expression in single-cell RNA-seq studies [61].

### 3.4. Relationship of Differential Gene Expression and Chromatin Accessibility

**Figure 6:**
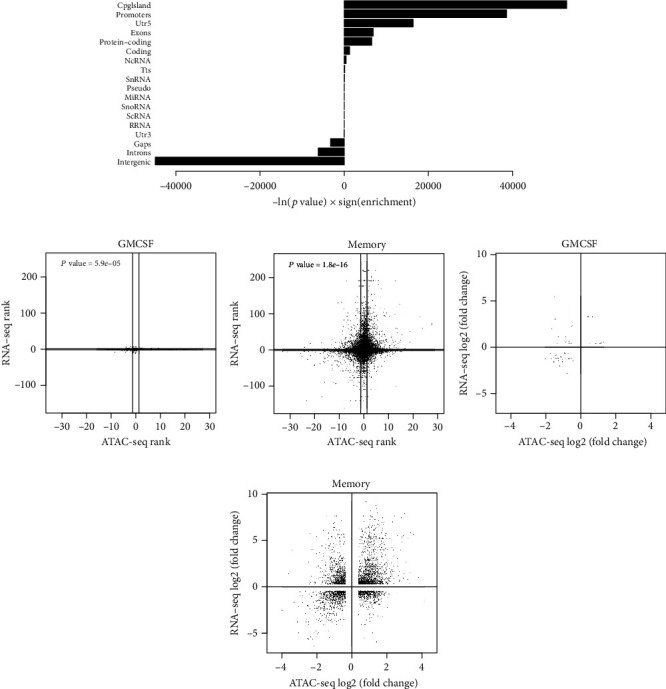
Enrichment of ATAC-seq peaks in regions representing certain genomic features and relationship of ATAC-seq and RNA-seq data. (a) The enrichment of ATAC-seq consensus peaks in different types of genomic regions is shown with bars representing the enrichment significance × direction of enrichment (−ln(*P* value) × sign(enrichment)). (b, c) Genes that were detected on the RNA-seq level and were also assigned to ATAC-seq peaks were ranked using the –log_10_(FDR) × sign(log_2_(fold change)) function based on both RNA-seq and ATAC-seq (using values of the assigned peaks) within a given cell type comparison ((b) GM-CSF+ *vs.* GM-CSF−; (c) memory *vs.* naïve). The ranks of genes using ATAC-seq *vs.* RNA-seq for ranking are visualized for both cell type comparisons; lines indicate FDR cutoff 0.05 in the respective comparison. *P* value represents the probability of observing this or more directional agreement between the two data types by chance, using only genes significantly differential (FDR < 0.05) in both data types. *P* values were calculated by Fisher's exact test with Monte Carlo simulation. (d, e) Dot plot representation as in (b, c), but using log_2_(fold change) for ranking and visualizing only (assigned) genes that are significantly differential in both data types.

Since chromatin accessibility can directly affect gene expression, we next combined the ATAC-seq and RNA-seq data, aiming to identify TFs that may bind to open chromatin regions and hence affect the expression of their target genes. The methods for calling consensus peaks are not trivial. Therefore, we first confirmed that the genes assigned to the consensus peaks defined in this study were enriched for several pathways involved in immune regulation including T cell receptor signaling and Th subset differentiation, as well as for immune-related diseases including MS, RA, and other autoimmune diseases (Supplementary Table S2A, B). Furthermore, the distribution of the consensus peaks (open chromatin regions) defined in our data showed an enrichment for being located in CpG islands, promoters, 5′ UTRs, exons, and protein-coding regions (Figure 6(a)), suggesting that the ATAC-seq data generated here should be well suited to identify TF binding and the expression of corresponding target genes. We first studied the relationship of RNA and chromatin data on a global level: We considered the genes that were both detected on the RNA level and also had an ATAC-seq peak assigned to them. We ranked these genes using the –log_10_ (FDR) × sign (log_2_ (fold change)) function separately for the RNA-seq and ATAC-seq data. Next, we visualized the correlation between these two ranks for each contrast (Figures 6(b) and 6(c)). Considering only the genes significantly changing (FDR < 0.05) in the given contrast and assigning them to up- and downregulated categories, we detected more than random coincidence in the direction of the change between the RNA-seq and ATAC-seq data (using Fisher's exact test with Monte Carlo simulation; Figures 6(b) and 6(c)). Increased openness of the chromatin was associated with increased expression of the corresponding gene's RNA for the majority of genes. Less accessible chromatin also coincided with low expression of the corresponding gene in many cases, although a substantial fraction of lowly accessible regions also displayed high expression of the corresponding gene (Figures 6(d) and 6(e)).

### 3.5. Identification of Key TFs Linked to the Signatures of GM-CSF-Positive and Memory CD4 T Cells

**Figure 7:**
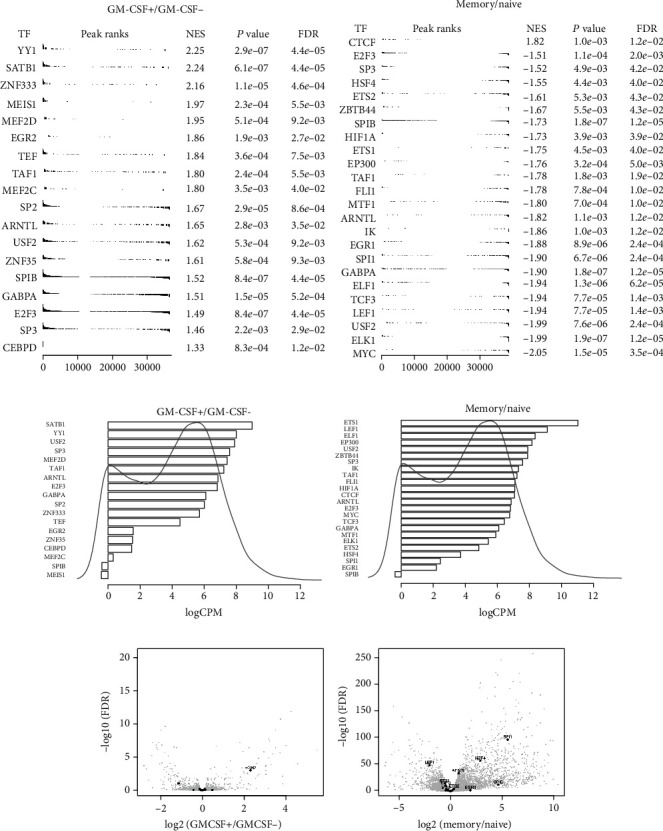
Identification of key TFs with global regulatory effect. (a, b) Rank-based enrichment analysis of TF binding motifs in footprints within peaks that were ranked based on differential accessibility using the –log_10_(FDR) × sign(log_2_(fold change)) function. The analysis was performed separately for the GM-CSF+/GM-CSF− and memory/naïve cell comparison ((a) and (b), respectively). NES: normalized enrichment score. (c, d) Average expression levels of identified key TFs for both cell comparisons are shown as log(count per million reads). To indicate the relative expression level of these TFs (bars) in view of the spectrum of lowly and highly expressed genes, the scaled kernel density estimate is shown based on the distribution of the average expression of all genes. (e, f) Volcano plots show the differential expression (effect size *vs.* significance) of the TFs identified as key regulators in each of the cell type comparisons (GM-CSF+/GM-CSF− and memory/naïve cell comparison; (e) and (f)). Key TFs are indicated as black dots, and those which are DEGs are labeled with their gene symbol.

To identify potential TFs that may establish the gene signatures of GM-CSF-positive (versus GM-CSF-negative) and memory (versus naïve) CD4 T cells, we scanned the consensus peaks for footprints, and subsequently, we scanned the identified footprints for TF binding motifs. For motif scanning, we used the TRANSFAC database [47] that contains experimentally validated binding sites, consensus binding sequences (positional weight matrices), and regulated genes of eukaryotic TFs. Confirming the methodology used to identify footprints, they were enriched on the expected ends of the ranked peak lists for the corresponding cell type comparisons (Supplementary Figure S5). Next, we defined the TFs whose binding sites were most enriched in peaks of GM-CSF-positive or memory cells (ranked based on differential accessibility) and identified about 20 TFs each that passed the significance threshold (FDR < 0.05) for enrichment (Figures 7(a) and 7(b)). These lists contained several TFs with a well-known role in T cells, such as SATB1, YY1, ETS, and EGR family TFs, among other factors with a less defined role. Notably, there was only little overlap between the key TFs in GM-CSF-positive and memory cells, suggesting that our strategy may have identified key factors to specifically define the GM-CSF-positive T cell phenotype. The majority of these TFs were highly expressed on the RNA level (Figures 7(c) and 7(d)), falling within the class of highly expressed genes (HEGs) that were suggested to be more likely to be functional than lowly expressed genes [50]. Interestingly however, most of these TFs were not differentially expressed themselves in the cell population comparisons under study (Figures 7(e) and 7(f)), suggesting that they may be regulated on posttranscriptional levels such as protein phosphorylation and intracellular localization which is well known for many TFs. Hence, with a strategy exploring solely the transcriptome (or even proteome) without integrating ATAC-seq data, several key TFs would likely be missed, while our integrative strategy combining RNA-seq and ATAC-seq data successfully identified such TFs from limited amounts of primary human T cells.

### 3.6. Integration of RNA-Seq and ATAC-Seq Data Identifies Gene Regulatory Networks of GM-CSF-Positive and Memory CD4 T Cells

Having identified key TFs in GM-CSF-positive and memory CD4 T cells, we were interested whether these factors regulated certain groups of target genes and whether several TFs may act together in a concerted fashion. Some TFs regulated a large number of target genes, and clusters of TFs were grouping together (Supplementary Figure S6A, B). Exploring the cobinding between TFs in more detail showed that certain groups of TFs bound together to the same targets (Supplementary Figure S6C, D). These included the TCF3:LEF1 pair that was always cobinding the same regions, as evident from the memory/naïve CD4 T cell contrast (Supplementary Figure S6B, D). TCF/LEF family proteins act downstream of the Wnt pathway, and it is well known that they often display overlapping expression patterns and functional redundancy [74]. In accordance with our data on human memory and naïve CD4 T cells, binding motifs for TCF family members were recently also determined to be depleted in murine memory CD8 T cells as well as human memory CD4 T cells using ATAC-seq [35, 39].

**Figure 8:**
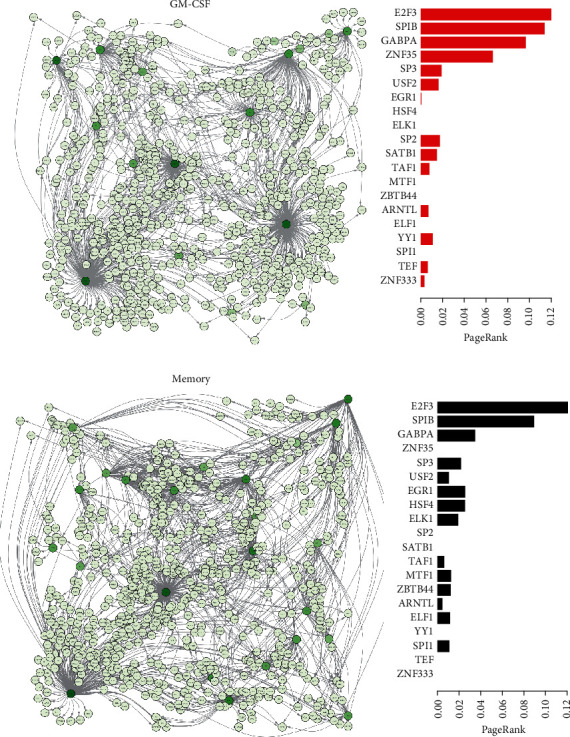
Gene regulatory network of GM-CSF+/GM-CSF− or memory/naïve CD4 T cells. (a, b) Gene regulatory network reflecting the signatures of GM-CSF-positive versus GM-CSF-negative CD4 T cells. (a) A directed network representing binding from source TFs to target peaks assigned to the indicated gene is shown. Source nodes were selected as described (Figure 7, key TFs), and target nodes were selected to be either differentially accessible (as a peak assigned to the respective node gene) and/or to be differentially expressed on the mRNA level in the GM-CSF+/GM-CSF− contrast. To calculate the PageRank [51] as a measure of importance of the TFs (with TFs influencing more genes being more important), the edges were first inverted (not displayed), and the computed PageRank value is represented by the color scale (light to dark green for lower to higher PageRank). (b) Those TFs having a PageRank value higher than the 99-percentile in either the GM-CSF and/or the memory cell network and their corresponding PageRank value in the GM-CSF network are displayed. (c, d) Same as (a, b), but for the memory/naïve CD4 T cell contrast.

Finally, by connecting the TFs to the genes assigned to their target regions, we generated a directed gene regulatory network representing GM-CSF-positive (versus negative) and memory (versus naïve) CD4 T cells, respectively (Figure 8). The source nodes were represented by the key TFs as selected above (key TFs from Figures 7(a) and 7(b)), and target nodes were selected when the region was either differentially accessible and/or the mRNA of the assigned gene was differentially expressed in the respective cell population comparisons. Both the GM-CSF and the memory comparisons led to similarly sized networks; however, only some of the target and source nodes were shared between both networks (Figures 8(a)–8(d)).

To quantify the importance of the source nodes in each individual network and to enable comparison of importance between both networks, we calculated scores based on the PageRank algorithm [51]. To do such a calculation on a network where the outgoing edges from a TF reflect its importance, rather than the incoming edges like in the original application of the algorithm, the edges in our networks were inverted (solely for the purpose of calculating the PageRank values). The obtained PageRank values give information on the importance of a node based on the number of other nodes it influences (directly or indirectly). Some of the key TFs that were shared between both networks also had a relatively high PageRank in both networks, such as the TFs E2F3, SPIB, and GABPA (Figures 8(a)–8(d)), and may be general regulators of T cell activation or differentiation. Importantly, we also identified key TFs with a relatively high PageRank in the GM-CSF T cell regulatory network whose PageRank value in the memory/naïve network was 0. These TFs, namely, ZNF35, SP2, SATB1, YY1, TEF, and ZNF333, may, thus, be novel regulators specifically controlling the molecular signatures of GM-CSF-positive CD4 T cells (Figures 8(a)–8(d)).

## 4. Conclusions and Future Perspectives

In summary, we here provide interpreted high-quality novel RNA-seq and ATAC-seq data from primary human CD4 T cells, with a focus on GM-CSF-positive cells that are known to play an important role in the autoimmune disease MS. We provide a network of key TFs and their targets representing GM-CSF-positive cells and memory CD4 T cells. To our knowledge, this is the first study providing signatures of GM-CSF-secreting cells isolated by capture assay without restimulation, which are likely as similar as currently possible to the state *in vivo* in humans. Recently, others have used a similar approach, capturing IFN-*γ*, IL-17, and IFN-*γ*/IL-17 double-positive cells from human donors by cell sorting [75]. Although the authors used restimulation with PMA and ionomycin for 3 hours, the data are especially interesting as they include samples from MS patients [75]. The authors studied only a limited set of 418 genes in the captured IFN-*γ*- and IL-17-secreting cells, and unfortunately, they did not include GM-CSF-producing cells, while the methods employed in the present work enable genome-wide studies of DNA accessibility and RNA expression patterns in these cells. Nevertheless, the authors obtained important results on the transcriptional signatures of IFN-*γ*- and IL-17-secreting cells in MS patients, including genes distinguishing clinically stable versus active MS patients. This raises interesting prospects for studying GM-CSF-positive cells specifically from MS patients as well by the methods and comparing to the data resource provided here. This may have important clinical implications given the likely important contribution of GM-CSF-positive CD4 T cells to MS. Future work should also address what distinguishes patterns and contributions of GM-CSF versus IFN-*γ*- and IL-17-producing T cells, as well as those coproducing GM-CSF and IFN-*γ*, particularly in MS patients, and ideally even from the affected tissue such as cerebrospinal fluid. However, this is technically challenging due to limitations in the amount of sample. Furthermore, analysis of cytokine producing cells, unfortunately, usually requires artificial restimulation or lengthy cell isolation procedures that may affect the gene signature drastically. Even in our dataset where we avoided artificial restimulation with PMA and ionomycin, dimensionality reduction analysis revealed that the capture assay and column procedure by itself had an effect on the transcriptome, as GM-CSF-positive and negative cells appeared more similar to each other than the GM-CSF-negative cells were to bulk CD4 T cells (which should largely overlap and which also underwent a negative selection for CD4 T cell isolation). On the contrary, ATAC-seq data seemed more robust against such effects, suggesting that the epigenetic signature may be especially suited to reflect *in vivo* patterns less influenced by procedures related to cell sample preparation. Hence, studies on cytokine-producing Th subsets from MS patients should in the future be extended to ATAC-seq and to the GM-CSF-producing Th subset. Interestingly, recent ATAC-seq data from murine GM-CSF versus IFN-*γ*- and IL-17-producing T cells upon *in vitro* stimulation suggests that while all subsets showed distinct epigenetic profiles, GM-CSF-producing cells were more related to IFN-*γ*- than to IL-17-producing T cells [10]. With an elegant fate mapping system, that study [10] could also demonstrate that murine GM-CSF-producing T cells display a stable epigenetic imprint. While such fate mapping studies are not possible with human cells, our study provides data from *ex vivo*-isolated human cells that may be most relevant for human diseases and it is tempting to speculate that such epigenetic signatures could be reflected in T cells isolated from MS patients. Together, our data on naïve, memory, and GM-CSF-positive CD4 T cells (and corresponding controls) can be exploited for a multitude of future studies for basic and translational immunology concerning autoimmune diseases. Beyond that, the results may have important implications for other diseases with involvement of GM-CSF-producing cells, such as sepsis and COVID-19. Further, our data can be a testbed for bioinformatics method development for the integration of RNA-seq and ATAC-seq data from the same samples, which may help understanding the basic principles of gene regulation in primary eukaryotic cells.

## Data Availability

The datasets are available in the GEO repository under the SuperSeries accession number GSE119734 (with the SubSeries GSE119731 and GSE119732).

## Supplementary Materials

Supplementary Materials contain the following Figures and Tables: Supplementary Table S1A: disease enrichment analysis based on the gene list ranked by gene expression in GM-CSF-positive CD4 T cells (versus GM-CSF-negative CD4 T cells). Supplementary Table S1B: disease enrichment analysis based on the gene list ranked by gene expression in memory (versus naïve) CD4 T cells. Supplementary Table S2A: KEGG pathway enrichment analysis of genes assigned to consensus peaks. Supplementary Table S2B: GWAS catalog disease enrichment analysis of genes assigned to consensus peaks. Supplementary Figure S1: purity of cell populations used in this study. Supplementary Figure S2: expression profile and DNA accessibility signatures of human memory CD4 T cells. Supplementary Figure S3: comparison of published and novel naïve and memory T cell transcriptome data. Supplementary Figure S4: cytokine coexpression in GM-CSF-producing T cells. Supplementary Figure S5: enrichment of footprints across peaks ranked based on differential accessibility. Supplementary Figure S6: network structure identifying shared TF binders across peaks and shared targets of key TFs.
