# Supplementary material for: Gene Regulatory Network of Human GM-CSF-Secreting T Helper Cells
Source: J Immunol Res. 2021 Jul 3;2021:8880585. doi: 10.1155/2021/8880585 (PMC8275380; doi:10.1155/2021/8880585)
Supplement: Supplementary Materials — contain the following Figures and Tables: Supplementary Table S1A: disease enrichment analysis based on the gene list ranked by gene expression in GM-CSF-positive CD4 T cells (versus GM-CSF-negative CD4 T cells). Supplementary Table S1B: disease enrichment analysis based on the gene list ranked by gene expression in memory (versus naïve) CD4 T cells. Supplementary Table S2A: KEGG pathway enrichment analysis of genes assigned to consensus peaks. Supplementary Table S2B: GWAS catalog disease enrichment analysis of genes assigned to consensus peaks. Supplementary Figure S1: purity of cell populations used in this study. Supplementary Figure S2: expression profile and DNA accessibility signatures of human memory CD4 T cells. Supplementary Figure S3: comparison of published and novel naïve and memory T cell transcriptome data. Supplementary Figure S4: cytokine coexpression in GM-CSF-producing T cells. Supplementary Figure S5: enrichment of footprints across peaks ranked based on differential accessibility. Supplementary Figure S6: network structure identifying shared TF binders across peaks and shared targets of key TFs. [file 8880585.f1.zip › Elias_et_al_Supplementary_Figures (1).pdf]

Supplementary Figure S1

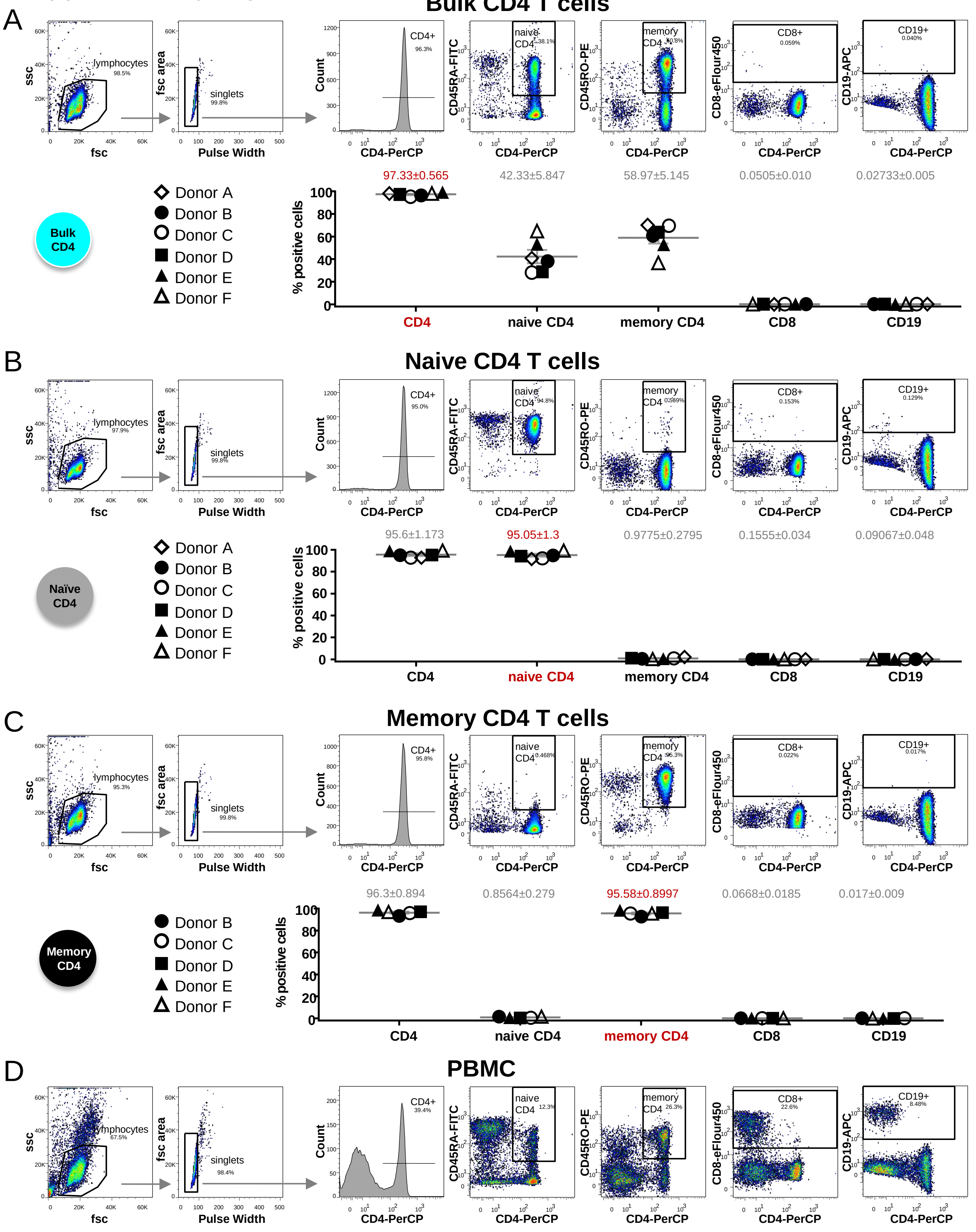

Supplementary Figure S1 (continued)

Supplementary Figure S1: Purity of cell populations. Total CD4 T cells (**A**), naive CD4 T cells (**B**) and memory CD4 T cells (**C**) were isolated in parallel by negative magnetic isolation from human PBMCs. Cells were stained for the indicated surface markers and analyzed by flow cytometry. Upper panels show flow cytometry raw data for a representative donor each. The two scatterplots to the left show the pre-gating strategy; the 5 flow cytometry plots to the right are pre-gated on doublet-excluded lymphocytes. Gates to determine percentages of CD4+ cells, naive CD4+ cells, memory CD4+ cells, CD8+ cells and CD19+ cells are indicated. Lower panels show the summary data for the gates defined as above, for n=5 to 6 donors from 4 independent experiments. Same symbols (filled or unfilled) indicate donors processed within the same experiment. Horizontal grey lines represent mean values, and error bars in grey represent SEM. Mean±SEM values are indicated in grey, or highlighted in red font for the most relevant gate for the given isolated cell population. (**D**) shows PBMCs from a representative donor, which were always included in the staining as positive control. Mean±SEM values for PBMCs (n=6 donors) were as follows. CD4+ cell gate: 45.60±3.4%; naive CD4+ cell gate: 19.75±3.6%; memory CD4+ cell gate: 25.53±2.98%; CD8+ cell gate: 22.15±2.44 %; CD19+ cell gate: 7.91±0.64%.

Supplementary Figure S2

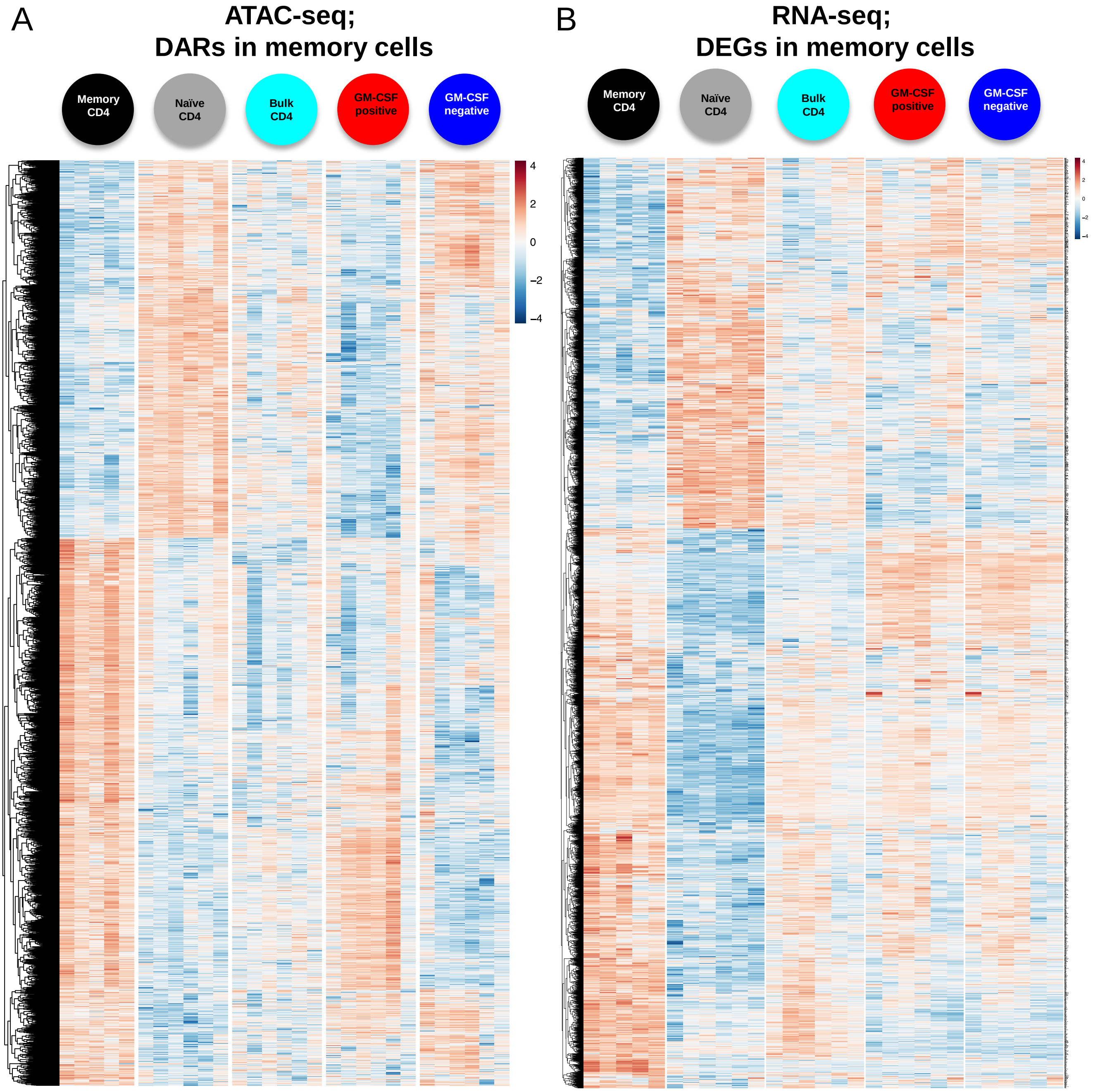

Supplementary Figure S2: (**A**) Differentially accessible DNA regions (DARs) in memory *versus* naive CD4 T cells are plotted as a heatmap for their accessibility in all the five cell populations studied. $\log_2$(RPKM+1) is represented by the color scale, indicating accessibility (blue: low, red: high). Data were row-scaled and -clustered (Euclidean distance complete linkage). (**B**) Differentially expressed genes (DEGs) in memory *versus* naive CD4 T cells are plotted as a heatmap for their expression in all the five cell populations studied. Gene expression is displayed as $\log_2$(FPKM+1) with blue indicating low and red indicating high expression according to the color scale. Data were row-scaled and -clustered (Euclidean distance complete linkage).

Supplementary Figure S3

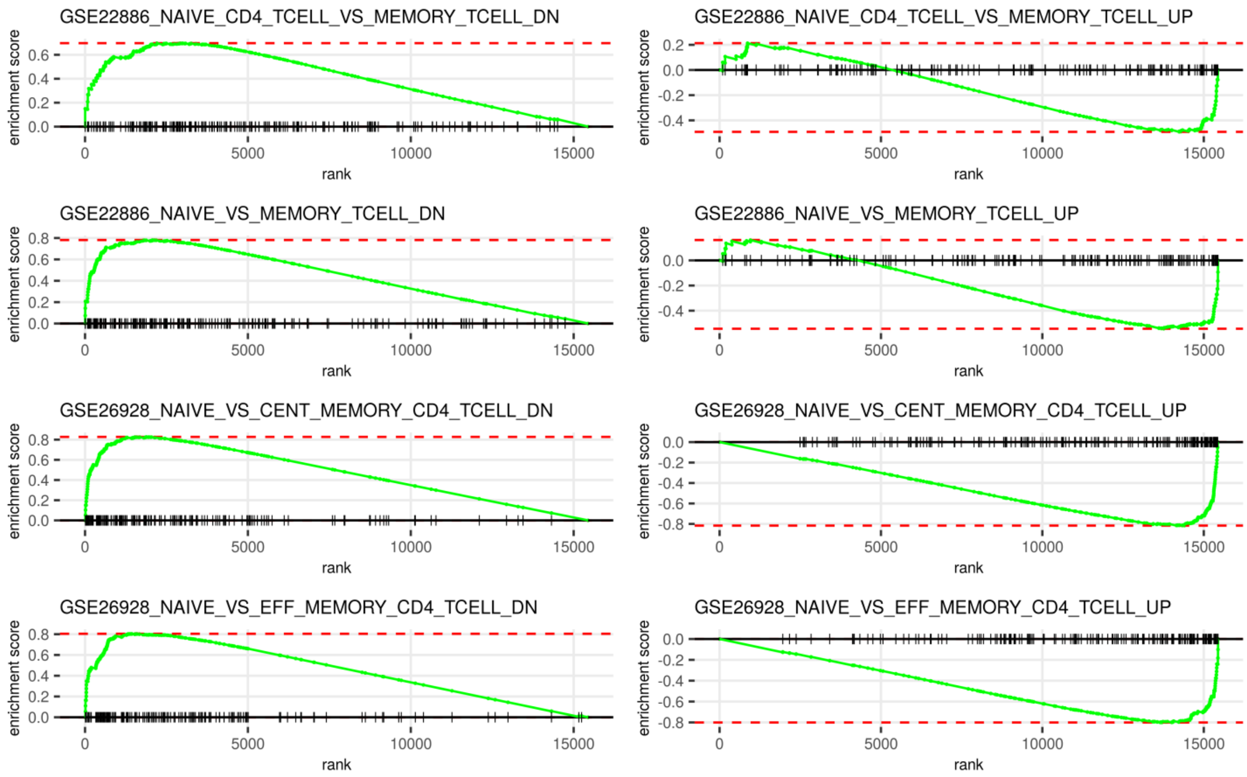

Supplementary Figure S3: Comparison of published and novel naïve and memory T cell transcriptome data. Enrichment analysis of genes from published transcriptome data sets featuring naïve and memory T cell subsets (GSE accession numbers as displayed) in a ranked gene list of our memory *versus* naïve T cell RNA-seq data. Enrichment of up- or downregulated genes from the GSE data sets across genes ranked based on differential expression in memory *versus* naïve T cells in our data was analyzed. Genes were ranked based on their differential expression in memory *versus* naïve T cells using the $-\log_{10}$(P value) $\times$ sign($\log_2$(Fold Change)). Genes are represented on the horizontal axis and the enrichment score is represented on the vertical axis. Gene sets containing both human naïve and memory T cell data were retrieved from the MSigDB database. Corresponding enrichment scores along with P values and FDRs are also displayed in Figure 3E.

Supplementary Figure S4

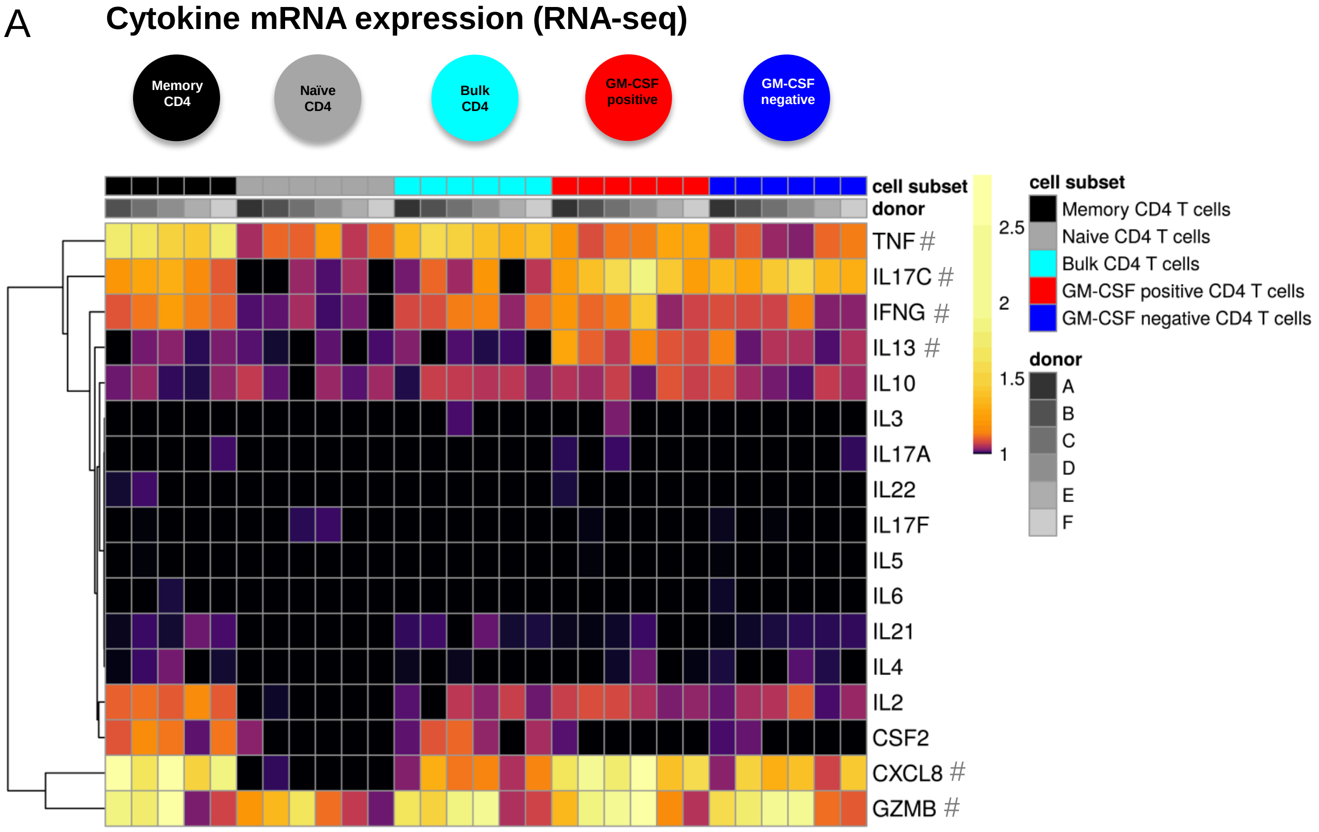

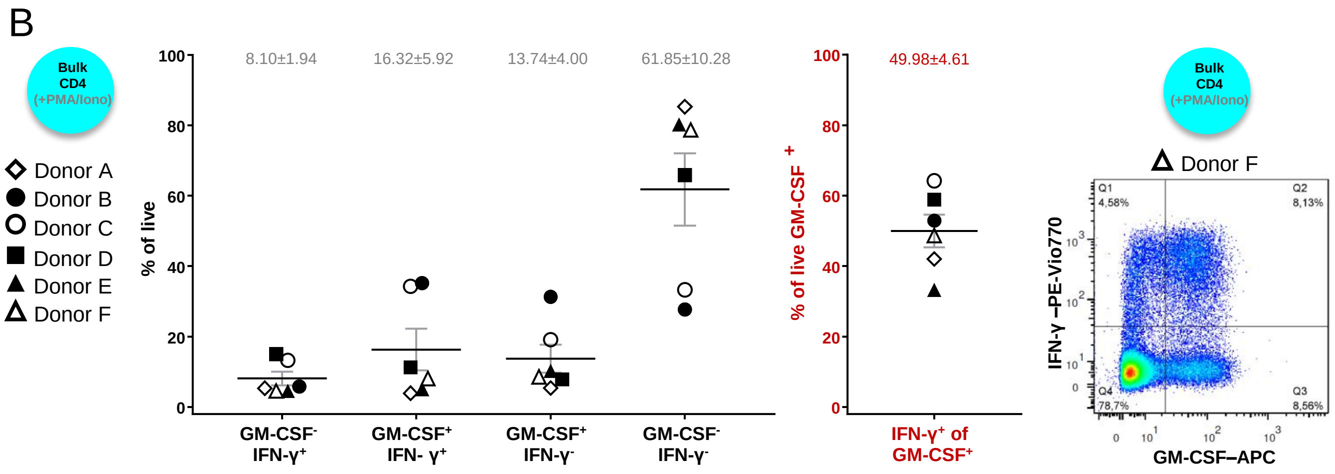

Supplementary Figure S4 (continued)

C

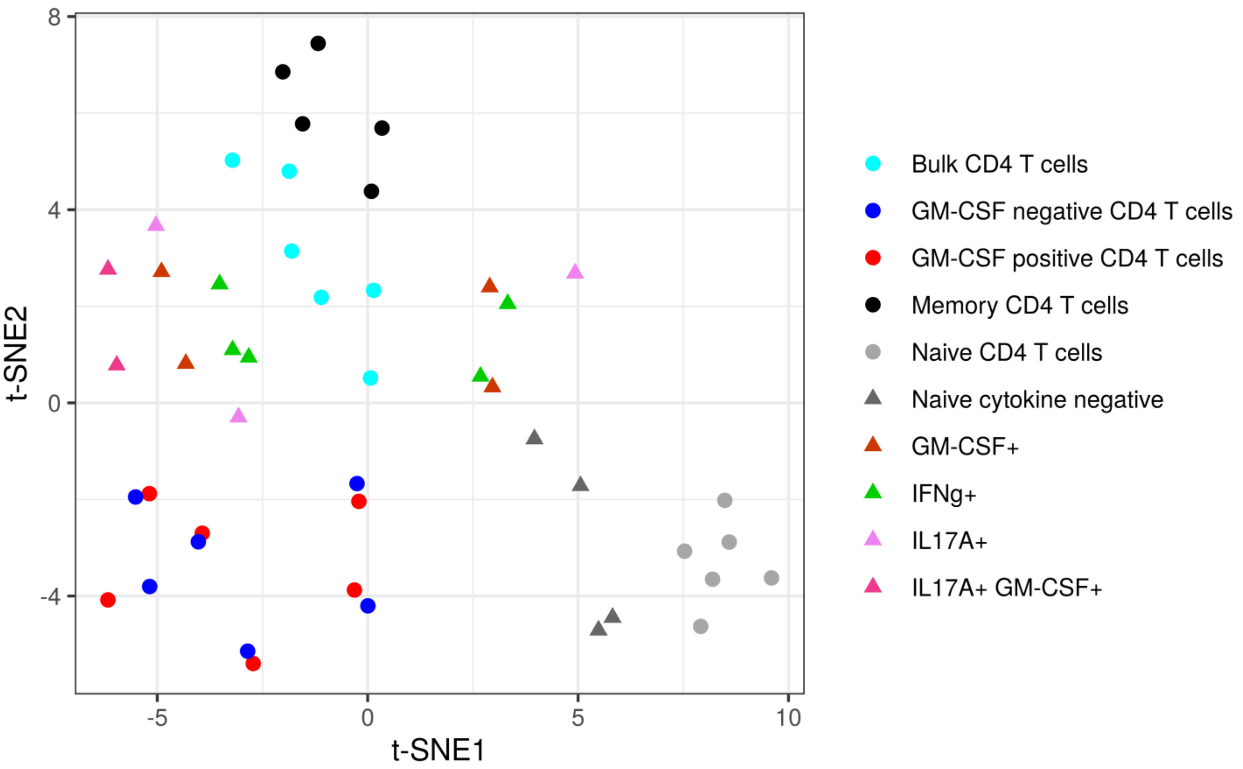

Supplementary Figure S4: Cytokine co-expression in GM-CSF producing T cells. (**A**) mRNA expression of cytokine genes in the different cell populations studied by RNA-seq. Donor and cell type are indicated on the top. Normalized counts per million (using FPKM normalization and log_2 transformation) are displayed by the color scale, and black cells represent samples without detected counts (count = 0). Data were row-clustered (Euclidean distance complete linkage). Only genes labeled with a " # " passed the detection limit (defined as more than 1 count per million in at least 3 samples) and were included in downstream differential expression analysis. (**B**) An aliquot of bulk CD4 T cells from the samples used for RNA-seq and ATAC-seq was taken and restimulated with 375 ng/ml ionomycin and 10 ng/ml PMA in the presence of GolgiPlug Protein Transport Inhibitor (containing Brefeldin A) for 4 hours at 37°C, 5% CO_2. Afterwards, cells were stained with fixable viability dye, fixed, permeabilized, and stained intracellularly with antibodies against GM-CSF and IFN-γ. Left panel: Fractions of GM-CSF and IFN-γ single or double positive cells (% of live cells) are shown. Middle panel shows the fractions of IFN-γ positive cells within GM-CSF positive cells. Right panel: raw data from a representative donor (pre-gated on live lymphocytes) are shown. (**C**) Transcriptome data from this study for the cell populations indicated by the colored dots were compared to transcriptome data from Al-Mossawi MH *et al.* (*Nature Communications*, 2017) for cell populations given by the colored triangles. Note that the cell populations in Al-Mossawi *et al.* were all subject to stimulation with PMA and ionomycin and therefore, to increase comparability between the studies, batch normalization using the ComBat method (Leek JT *et al.*, *Bioinformatics*, 2012) was applied before performing dimensionality reduction analysis by t-SNE.

Supplementary Figure S5

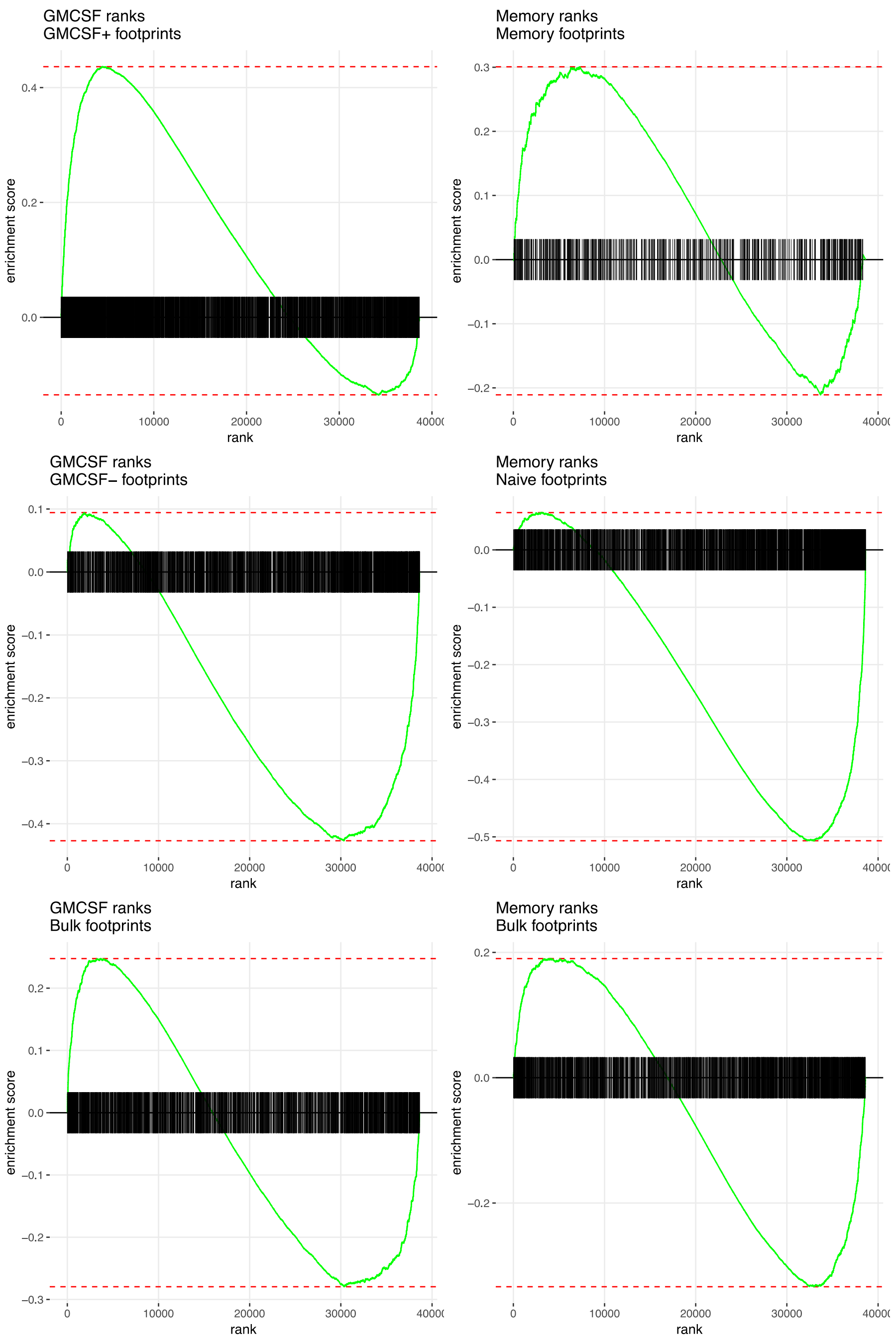

Supplementary Figure S5: Enrichment of footprints across peaks ranked based on differential accessibility. Peaks were ranked based on their differential accessibility in the indicated contrast using the $-\log_{10}(\mathrm{FDR}) \times \mathrm{sign}(\log_2(\mathrm{Fold\ Change}))$. Peaks are represented on the horizontal axis and the enrichment score of the set of footprints identified in the indicated cell population is represented on the vertical axis.

Supplementary Figure S6

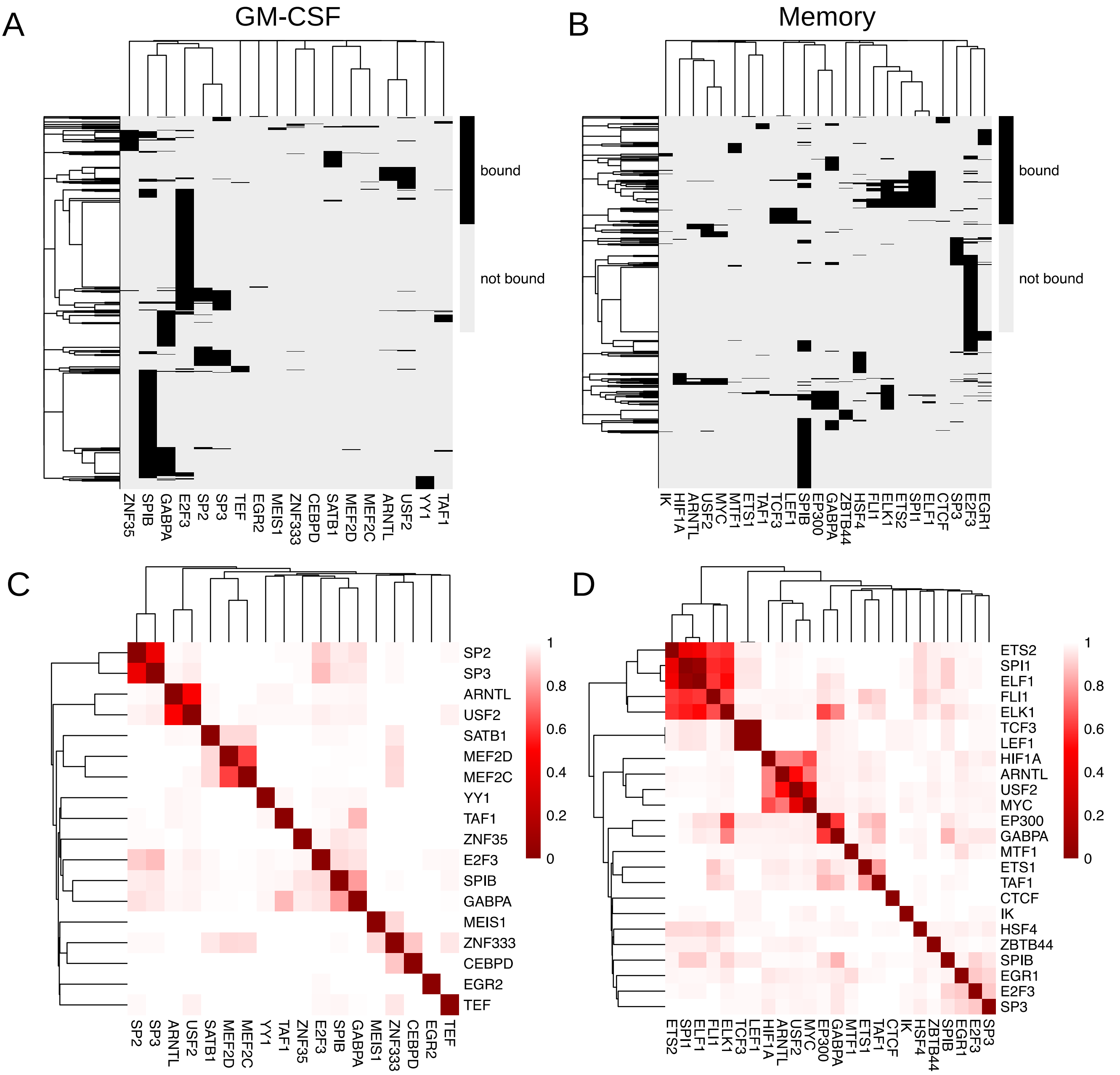

Supplementary Figure S6: Network structure identifying shared TF binders across peaks and shared targets of key TFs. (**A, B**) Key TFs were identified as in Figure 7. The binary heatmap shows the binding of key TFs (columns) in footprints within ATAC-seq peaks (rows) that are either differentially accessible and/or that are assigned to a differentially expressed gene in the GM-CSF+/GM-CSF- cell comparison (A) or in the memory/naïve cell comparison (B). Black indicates that the key TF binds to the respective region. Rows and columns are clustered using binary distance and complete linkage. (**C, D**) Heatmap representation of the binary distance matrix of the key TFs using the binary vector of binding (“bound” or “not bound”) for distance calculation. The red color scale represents the distance; smaller distance represents more co-binding. Rows and columns are clustered using Euclidean distance and complete linkage. The analysis was performed for the GM-CSF+/GM-CSF- cell comparison (C) or the memory/naïve cell comparison (D).
